# Long-term nitrogen fertilization of paddy soil shifts iron-reducing microbial community revealed by RNA-^13^C-acetate probing coupled with pyrosequencing

**DOI:** 10.1038/ismej.2014.159

**Published:** 2014-08-29

**Authors:** Long-Jun Ding, Jian-Qiang Su, Hui-Juan Xu, Zhong-Jun Jia, Yong-Guan Zhu

**Affiliations:** 1 State Key Laboratory of Urban and Regional Ecology, Research Center for Eco-Environmental Sciences, Chinese Academy of Sciences, Beijing, China; 2 University of Chinese Academy of Sciences, Beijing, China; 3 Key Laboratory of Urban Environment and Health, Institute of Urban Environment, Chinese Academy of Sciences, Xiamen, China; 4 State Key Laboratory of Soil and Sustainable Agriculture, Institute of Soil Science, Chinese Academy of Sciences, Nanjing, China

## Abstract

Iron reduction is an important biogeochemical process in paddy soils, yet little is known about the microbial coupling between nitrogen and iron reduction. Here, we investigated the shift of acetate-metabolizing iron-reducers under long-term nitrogen fertilization using ^13^C-acetate-based ribosomal RNA (rRNA)-stable isotope probing (SIP) and pyrosequencing in an incubation experiment, and the shift of putative iron-reducers in original field samples were investigated by 16S rRNA gene-based pyrosequencing. During SIP incubations, in the presence of iron(III) oxyhydroxides, more iron(II) formation and less methane production were detected in nitrogen-fertilized (N) compared with non-fertilized (NF) soil. In ^13^C-rRNA from microcosms amended with ferrihydrite (FER), *Geobacter* spp. were the important active iron-reducers in both soils, and labeled to a greater extent in N (31% of the bacterial classified sequences) than NF soils (11%). Pyrosequencing of the total 16S rRNA transcripts from microcosms at the whole community level further revealed hitherto unknown metabolisms of potential FER reduction by microorganisms including *Pseudomonas* and *Solibacillus* spp. in N soil, *Dechloromonas*, *Clostridium*, *Bacillus* and *Solibacillus* spp. in NF soil. Goethite (GOE) amendment stimulated *Geobacter* spp. to a lesser extent in both soils compared with FER treatment. *Pseudomonas* spp. in the N soil and *Clostridium* spp. in the NF soil may also be involved in GOE reduction. Pyrosequencing results from field samples showed that *Geobacter* spp. were the most abundant putative iron-reducers in both soils, and significantly stimulated by long-term nitrogen fertilization. Overall, for the first time, we demonstrate that long-term nitrogen fertilization promotes iron(III) reduction and modulates iron-reducing bacterial community in paddy soils.

## Introduction

Iron (Fe) is the most abundant redox-active element on the Earth, thus microbial iron redox cycling has a fundamental role in environmental biogeochemistry (Weber *et al.*, 2006a). Dissimilatory ferric iron [Fe(III)] reduction occurs under anoxic conditions when coupled to other biogeochemical processes, for instance the oxidation of organic matter or hydrogen (H_2_; Lovley *et al.*, 2004). The reduced ferrous iron [Fe(II)] can then be reoxidized under anoxic conditions when coupled to nitrite (NO_2_^−^) or nitrate (NO_3_^−^) reduction (Straub *et al.*, 1996), or to photosynthesis (Hegler *et al.*, 2008), or under microoxic conditions, regenerating oxidized Fe(III) to sustain Fe(III) reduction. Many studies have focused on dissimilatory Fe(III) reduction because of its global significance (Lovley *et al.*, 2004; Weber *et al.*, 2006a). It has been established that this metabolic process strongly influences carbon (C), nitrogen (N) and sulfur (S) cycling and indirectly affects nutrient availability, greenhouse-gas emissions and contaminant transformations (Borch *et al.*, 2010; Burgin *et al.*, 2011).

Microorganisms that mediate dissimilatory Fe(III) reduction are phylogenetically diverse (Lin *et al.*, 2007). Numerous dissimilatory iron-reducing microorganisms have been isolated, characterized and identified from paddy soils mainly by culture-dependent methods (Wang *et al.*, 2009; Li *et al.*, 2011). However, studies on the composition of microbial community participating in dissimilatory Fe(III) reduction have been rather limited because of the unavailability of universal functional gene markers. Stable isotope probing (SIP) analysis of ribosomal RNA (rRNA) is considered to be a powerful means to link the taxonomic identity of microorganisms to a specific function in complex environments (Manefield *et al.*, 2002; Vandieken and Thamdrup, 2013). It enables the identification of active acetate-oxidizing iron-reducing bacteria as a functional guild for the first time in an Italian anoxic paddy soil when amended Fe(III) oxyhydroxides were served as electron acceptors (Hori *et al.*, 2010). With the advent of high-throughput sequencing techniques, it is expected that the active communities of iron-reducers could be elucidated with an unprecedented detail and high-phylogenetic resolution.

Paddy soil represents an intermediate system between terrestrial ecosystems and aquatic ecosystems, and the alternation between oxic and anoxic conditions results in periodically occurring redox reactions. Owing to the unique characteristics of paddy soil and the elemental abundance of iron, dissimilatory Fe(III) reduction is prevalent and thought to be central to many other biogeochemical processes in flooded paddy soils (Yi *et al.*, 2013).

N fertilization is an important management practice for maintaining soil fertility and increasing rice yields in paddy soils (Cassman *et al.*, 1998), and believed to influence global biogeochemical processes, because of microbially mediated dissimilatory coupling between N and other elements such as C, Fe and S (Burgin *et al.*, 2011). Previous studies have shown that the abundance and community structure of some C-cycling and S-cycling communities such as methanogens (Mer and Roger, 2001), methanotrophs (Hanson and Hanson, 1996) and sulfate-reducers (Liu *et al.*, 2009) in paddy soils have altered under long-term N fertilization. Nevertheless, the effect of N fertilization on the composition of the microbial community responsible for dissimilatory Fe(III) reduction is unknown. Dissimilatory Fe(III) reduction has been found to be linked to N cycle in various environments such as wetland soils (Clément *et al.*, 2005), wastewater (Sawayama, 2006) and even tropical upland soils (Yang *et al.*, 2012). Therefore, we hypothesize that the dissimilatory iron-reducing microbial community alters after long-term N fertilizer (as urea) input in paddy soils.

The objective of this study was thus to investigate the active acetate-assimilating iron-reducing microbial community shifts under long-term N fertilization in a typical paddy soil from Southern China via an incubation experiment, in which two types of Fe(III) oxyhydroxides (that is, ferrihydrite (FER) and goethite (GOE)) were added as sole terminal electron acceptor and ^13^C-acetate-based rRNA-SIP in combination with recently developed high-throughput 16S rRNA pyrosequencing (454) techniques were used. Also, the shift of putative dissimilatory iron-reducers in original field samples were investigated by 16S rRNA gene-based pyrosequencing to examine the relevance between the results obtained from SIP incubations and those from field samples.

## Materials and methods

### Soil sampling

The long-term fertilization experiment site (established in 1990) was located in the Taoyuan Agro-ecosystem Research Station (28°55'N, 111°27'E), central Hunan Province of China; the field information is detailed in Supplementary Materials. In our study, surface soil samples (0–20 cm depth) were collected in September 2010 from the following two treatments: control without fertilizers (NF) and nitrogen fertilizer as urea (N). Each fertilization treatment had triplicate plots (33 m^2^ per plot), which were randomly arranged in the field, and all plots were sampled independently. Each soil sample was partitioned into two sub-samples: one was air dried, passed through a 2.0-mm sieve and stored at 4 °C for soil basic properties analyses and SIP incubations, and the second was stored at –80 °C for direct DNA extraction and 16S rRNA gene-based pyrosequencing. Soil chemical properties under these two fertilization treatments were analyzed by the standard methods (Lu, 1999), and listed in Table 1.

### SIP incubations and biogeochemical analyses

Before incubation, soil samples from triplicate plots within each fertilization treatment were mixed thoroughly, and then slurries were prepared as described previously (Hori *et al.*, 2007). To activate the soil microbes and deplete indigenous electron acceptors such as nitrate, available sulfate and Fe(III) (oxyhydr)oxides, the slurry was pre-incubated anoxically in the dark at 25 °C for 21 days (Hori *et al.*, 2010). Aliquots (5 ml) of the well-mixed slurry were placed into 25-ml serum vials. Three treatments were set up: (i) FER, (ii) GOE and (iii) control without any Fe(III) oxyhydroxide addition (CTR). Both Fe(III) oxyhydroxides were synthesized as described earlier (Schwertmann and Cornell, 1996), and added at a final concentration of 140 μmol g^−1^ soil dry weight. The μmol amount of FER added was calculated using the formula Fe_5_HO_8_ × 4 H_2_O (Kappler and Straub, 2005). Vials were then sealed with butyl rubber septa and crimped with aluminum caps, and headspaces were flushed with N_2_. Labeled experiments (*n*=3, each) were initiated by adding [U-^13^C]-labeled acetate (1, 2-^13^C_2_-acetate, 99 atom% Cambridge Isotope Laboratories, Andover, MA, USA) to each vial at a final concentration of 3.3 μmol g^−1^ soil dry weight (about 2 mM in pore water), and incubated statically for 4 days at 25 °C. Twice during the incubation (after 1.5 and 3 days), ^13^C-acetate was supplemented to the microcosms at a final concentration of 3.3 μmol g^−1^ soil dry weight. Thus, in total, 30 μmol of ^13^C-acetate were added to both soil slurries. In addition, parallel unlabeled experiments were established only for molecular analyses, and they were performed by the same procedure with the exception that the substrate was ^12^C-unlabeled acetate.

For the labeled experiments, the destructive sampling was performed in triplicate at days 0, 0.5, 1, 1.5, 2, 3 and 4. Total CH_4_ and CO_2_ in headspace samples of each vial were analyzed using a robotized sampling and analyzing system as described previously (Molstad *et al.*, 2007). The ^13^C atom percentage of CH_4_ and CO_2_ was measured by GC-isotope ratio mass spectrometry (Thermo Finnigan Delta V Advantage, Bremen, Germany) as described earlier (Conrad *et al.*, 2000). Pore water samples for volatile fatty acids analysis were taken, centrifuged and filtered as previously described (Krumböck and Conrad, 1991). Volatile fatty acids were determined by ion chromatography (Dionex ICS-3000 system, Dionex, Sunnyvale, CA, USA; Li *et al.*, 2011). Ferrous iron [Fe(II)] and total extractable Fe in soil slurry samples were measured as described by Achtnich *et al.* (1995). Briefly, about 0.5 g of slurry sample was pipetted into 4.5 ml of 0.5 M HCl for 24 h extraction, and extracted Fe(II) was determined using the ferrozine method. Total extractable Fe was analyzed by the same procedure with the exception that the extractant was 5-ml of 0.25 M hydroxylamine hydrochloride in 0.25 M HCl. The amount of hydroxylamine reducible Fe(III) was calculated as the difference between total extractable Fe and Fe(II) (Lovley and Phillips, 1987; Achtnich *et al.*, 1995). Furthermore, pH of soil slurries was determined using a pH meter (FE20, Mettler-Toledo, Zurich, Switzerland). The rest of the soil slurry samples were stored at –80 °C for subsequent molecular analyses.

### Soil slurry RNA extraction and SIP gradient fractionation

For both labeled and unlabeled experiments, RNA was extracted from soil slurry sample from each set of each treatment after 4 days of the incubation using the protocol of Griffiths *et al.* (2000) with the modification that glass beads was included in the lysis procedure, and this procedure was performed twice. The RNA extraction method is detailed in the Supplementary Materials. rRNA was purified by the RNeasy mini kit (Qiagen, Hilden, Germany) according to the manufacturer's instructions, and quantified using UV–vis Spectrophotometer (ND-1000, NanoDrop Technologies, Wilmington, DE, USA). Extracted rRNA (approximately 500 ng) was mixed well with cesium trifluoroacetate gradients to achieve an initial buoyant density (BD) of 1.790 g ml^−1^ before ultracentrifugation at 130 000 *g* for 65 h at 20 °C (Xia *et al.*, 2011). Centrifuged rRNA gradients were fractionated, the cesium trifluoroacetate BD of each fraction measured and rRNA precipitated from fractions as described previously (Lueders *et al.*, 2004a).

### Domain-specific PCR quantification of density-resolved rRNA

rRNA from each gradient fraction of each treatment was quantified in triplicate by real-time reverse transcription PCR in an iCycler iQ Thermocycler (Bio-Rad, Hercules, CA, USA) with primers Ba519f/Ba907r and Ar109f/Ar912rt targeting all *Bacteria* and *Archaea*, respectively (Lueders *et al.*, 2004a). Detection chemistry and thermal profiles were the same as described earlier (Lueders *et al.*, 2004a). Standardization of bacterial and archaeal templates was done as described by Lueders *et al.* (2004b).

### Terminal restriction fragment length polymorphism (T-RFLP) analysis of density-resolved rRNA

rRNA from each gradient fraction of each treatment was subjected to reverse transcription PCR for T-RFLP profiling. PCR primers used and amplification conditions were as reported earlier (Hori *et al.*, 2007); 25 and 30 cycles were performed for amplification of bacterial and archaeal templates, respectively. Amplicons were digested using *Msp*I and *Taq*I for *Bacteria* and *Archaea*, respectively. Digestion product was purified and size-separated as described previously (Cao *et al.*, 2012).

### 16S rRNA-based pyrosequencing

Selected density fractions of rRNA were reversely transcribed into complementary DNA (cDNA) using PrimeScript 1st strand cDNA synthesis kit (TaKaRa Biotech, Dalian, China) following the manufacturer's instructions, and subjected to pyrosequencing. The V4–V5 hypervariable regions of 16S rRNA genes were amplified using a primer set: 515f (5′-GTGCCAGCMGCCGCGG-3′) and 907r (5′-CCGTCAATTCMTTTRAGTTT-3′), containing the 454 FLX adapters and barcodes for sample identification (Zhou *et al.*, 2011). Each 50-μl reaction mixture contained 2 μl of template cDNA, 0.8 μM of each primer, 0.4 mM of each dNTP (TaKaRa Bio, Otsu, Japan), 5 μl of 10 × PCR buffer (Mg^2+^ plus; TaKaRa Bio), 1.5 U of *TaKaRa Taq* HS and 10 μg of BSA (TaKaRa Bio). Amplifications were performed using the thermal conditions as reported earlier (Xu *et al.*, 2014). After purification with Wizard sv gel and PCR clean-up system (Promega, Madison, WI, USA), equal amounts of the PCR products with different barcodes were mixed and submitted to the BGI Shenzhen (Shenzhen city, China) for pyrosequencing on a 454 GS FLX+ system.

### DNA extraction, amplification and 16S rRNA gene-based pyrosequencing in field samples

High-molecular weight community DNA was extracted from field samples from triplicate plots within each fertilization treatment, using the freeze-grinding, SDS-based method (Zhou *et al.*, 1996) and was purified by a low-melting agarose gel followed by phenol extraction. DNA quantity and purity were determined by UV–vis Spectrophotometer as described above. The purified DNA was amplified for pyrosequencing using a primer set: 577f (5′-AYTGGGYDTAAAGNG-3′) and 926r (5′-CCGTCAATTYYTTTRAGTTT-3′), containing the 454 FLX adapters and barcodes. This primer set also targeted the V4–V5 hypervariable regions of 16S rRNA genes (Yang *et al.*, 2014). PCR was conducted using the same conditions as for 16S rRNA-based pyrosequencing but with different primers. PCR products with different barcodes were purified and then mixed in equal amounts for pyrosequencing on a 454 GS FLX system.

### Processing of pyrosequencing data

Acquired raw pyrosequencing data were processed following the procedure described earlier (Xu *et al.*, 2014), using the Quantitative Insights Into Microbial Ecology toolkit-version 1.6.0 (Caporaso *et al.*, 2010). Briefly, after removing any low quality or ambiguous reads, qualified sequences were clustered into operational taxonomic units at 97% identity threshold. The most abundant sequence in the cluster for each operational taxonomic unit was chosen as a representative sequence for that operational taxonomic unit, and was assigned to taxonomy using the ribosome database project Classifier (version 2.2) with a minimum confidence threshold of 80% (Cole *et al.*, 2005).

For 16S rRNA-based pyrosequencing, beta diversity (UniFrac) analysis was conducted on a randomly chosen subset of 3990 sequences per sample to correct for unequal sequencing depth across samples.

For 16S rRNA gene-based pyrosequencing, following the taxonomic analysis at the genus level by ribosome database project Classifier, sequences related to putative dissimilatory iron-reducing bacteria were selected according to published reviews (Lovley *et al.*, 2004; Lovley, 2006; Weber *et al.*, 2006a) because of the absence of functional gene markers for dissimilatory iron-reducing microorganisms.

### Statistical analysis

Details of statistical analysis are given in the Supplementary Materials. SPSS (version 16.0, SPSS Inc., Chicago, IL, USA) software was used to perform standard statistical tests, including one-way and two-way analysis of variance, on the soil biogeochemical and taxonomic data.

For 16S rRNA-based pyrosequencing, differences in microbial communities among different rRNA fraction samples were analyzed using the phylogeny-based unweighted UniFrac distance metric (Lozupone and Knight, 2007). Average relative abundance data of predominant genus-level taxonomy in each treatment for each soil were transformed [log_2_(x+1)] and then served as input for the R PhyloTemp function to generate a heat map (Campbell *et al.*, 2010).

For 16S rRNA gene-based pyrosequencing, a non-parametric multivariate statistical test, adonis and canonical correlation analysis were performed using vegan package (version 2.0-8) (Oksanen *et al.*, 2013) in R version 3.0.0 (The R Foundation for Statistical Computing, Vienna, Austria).

### Accession number of nucleotide sequences

The 16S rRNA-based and 16S rRNA gene-based pyrosequencing reads have been deposited at GeneBank with accession number SRP033091 and SRP043656, respectively.

## Results

### Iron reduction in SIP incubations

**Figure 1:**
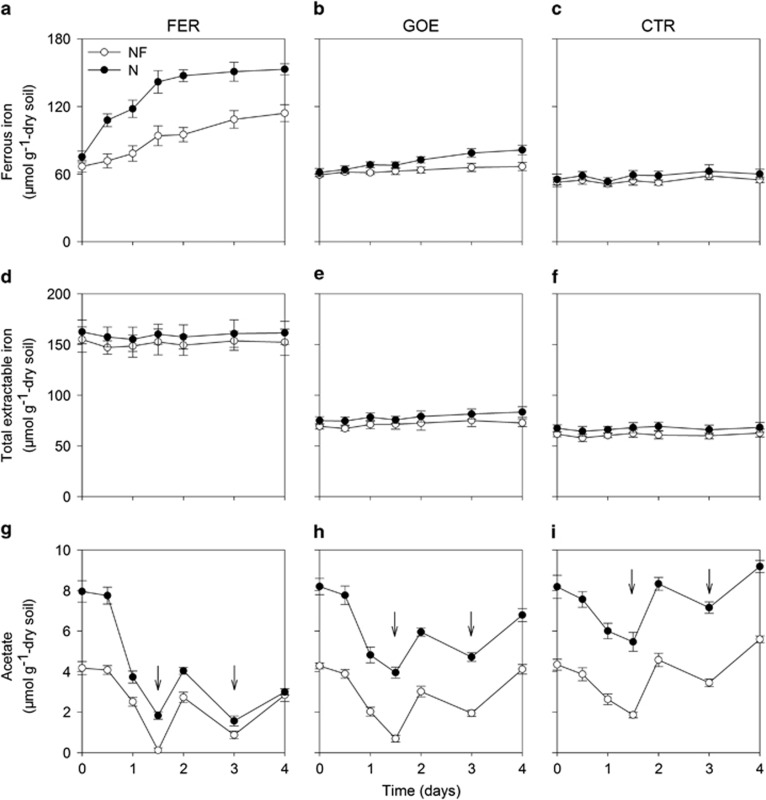
Time course of ferrous iron (**a**–**c**), total extractable iron (**d**–**f**) and acetate (**g**–**i**) during the anoxic incubations of non-fertilized (NF) and N-fertilized (N) paddy soil slurries in the ferrihydrite (FER; left panels **a**, **d**, **g**), goethite (GOE; middle panels **b**, **e**, **h**) and control (no ferric iron oxyhydroxide amended, CTR; right panels **c**, **f**, **i**) treatments. The arrows in **g**–**i** indicate that ^13^C-acetate was replenished to soil microcosms after days 1.5 and 3. The error bars represent the standard deviations of three replications.

For NF and N soils, Fe(II) was formed in the FER and GOE treatments, but was rarely detected in the CTR treatment (Figures 1a–c). In the presence of FER, Fe(II) concentration in the N soil increased markedly from 75 μmol g^−1^ at day 0 to 153 μmol g^−1^ at day 4, and the extent of increase was much larger than that in the NF soil (increased by 47 μmol g^−1^ after 4 days). Following the addition of GOE, a slight but significant (*P*<0.05) increase in Fe(II) concentration was observed after 4 days in both soils, and the extent of this increase in the N soil (by 20 μmol g^−1^) was also much higher than that in the NF soil (by 7.6 μmol g^−1^). Total extractable Fe in both soils treated with FER, GOE and CTR remained constant at about 155, 75 and 63 μmol g^−1^ throughout the incubation, respectively (Figures 1d–f).

### Acetate turnover in SIP incubations

During the first 1.5 days, acetate consumption in the FER treatment (4.1–6.1 μmol g^−1^) was significantly greater than in the GOE (3.6–4.3 μmol g^−1^) and CTR (2.5–2.7 μmol g^−1^) treatments in both soils (Figure 1g–i). Owing to almost complete degradation of the acetate in both soils treated with FER (approximately 77–97% degraded) within 1.5 days, ^13^C-acetate was replenished to the microcosms after 1.5 and 3 days of incubation. In total, acetate consumption in the N soil treated with Fe(III) oxyhydroxides (that is, FER and GOE) was 8.0–12 μmol g^−1^ after 4-day incubation, which was apparently larger than in the NF soil (6.8–7.9 μmol g^−1^). Other volatile fatty acids (for example, propionate or butyrate) were not observed during the incubation.

**Figure 2:**
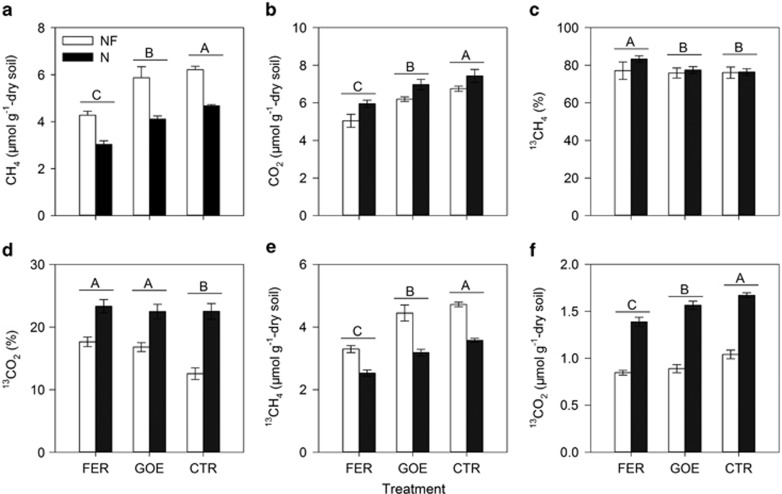
Changes of gaseous biogeochemical parameters in the non-fertilized (white bars; NF) and N-fertilized (black bars; N) paddy soil slurries treated with ferrihydrite (FER), goethite (GOE) and control (CTR) after 4-day incubation. (**a**–**f**) The concentrations of CH_4_ (**a**) and CO_2_ (**b**), ^13^C atom percentage of CH_4_ (**c**) and CO_2_ (**d**), the concentrations of ^13^CH_4_ (**e**) and ^13^CO_2_ (**f**) at the end of 4-day incubation. The different capital letters above the horizontal line denote significant differences among FER, GOE and CTR treatments at *P*<0.05 regardless of fertilization practices. The error bars represent the standard deviations of three replications.

Total CH_4_ production after 4 days was largely suppressed by up to 35% by the addition of FER (31–35%), but not much by GOE (5.5–12%) in both soils (Figure 2a). Furthermore, headspace CH_4_ concentration was significantly lower in all treatments in the N (3.0–4.7 μmol g^−1^) than NF soils (4.3–6.2 μmol g^−1^). Headspace CO_2_ accumulation in both soils was also significantly inhibited by FER (20–25%) and GOE (6.2–8.2% Figure 2b); whereas headspace CO_2_ concentration in all treatments in the N soil was much higher than that in the NF soil after 4 days.

To trace the fate of ^13^C-acetate, the ^13^C-atom percentage of headspace CH_4_ and CO_2_ was monitored throughout the incubation. After 4 days, the extent of increase in ^13^C atom percentage of CH_4_ and CO_2_ in the presence of Fe(III) oxyhydroxides was slightly but significantly (*P*<0.05) greater than that in the CTR treatment in both soils, respectively (Figures 2c and d). Furthermore, the ^13^C atom percentage of CH_4_ in the presence of Fe(III) oxyhydroxides (approximately 80%) revealed no significant difference between NF and N soils; in contrast, the ^13^C atom percentage of CO_2_ in the N soil treated with Fe(III) oxyhydroxides (up to 24%) was significantly higher than that in the NF soil (up to 18%). Overall, both headspace ^13^CH_4_ and ^13^CO_2_ concentrations were largely suppressed in the presence of FER, but not much by GOE in both soils (Figures 2e and f). In addition, compared with the NF soil, lower headspace ^13^CH_4_ concentration and higher headspace ^13^CO_2_ concentration were detected in the N soil in all treatments. These were consistent with the patterns obtained for total CH_4_ and CO_2_, respectively.

The mass of balance of the ^13^C-acetate added and gas products showed that 60 μmol of ^13^C from added acetate (2 × 30 μmol ^13^C-acetate) was converted to 16–22 μmol of ^13^CH_4_ plus ^13^CO_2_ in the gas and liquid phases in all treatments in both soils (Table 2). The ^13^C recovery in all treatments in both soils was only 27–36%.

### SIP of bacterial and archaeal 16S rRNA

rRNA-based SIP technique was applied in all treatments in both soils to trace microorganisms capable of ^13^C-acetate incorporation (defined as labeled treatments) after 4-day incubation. The incorporation of ^13^C into rRNA was corroborated by parallel incubation of microcosms with ^12^C-unlabeled acetate as the substrate (defined as unlabeled treatments).

**Figure 3:**
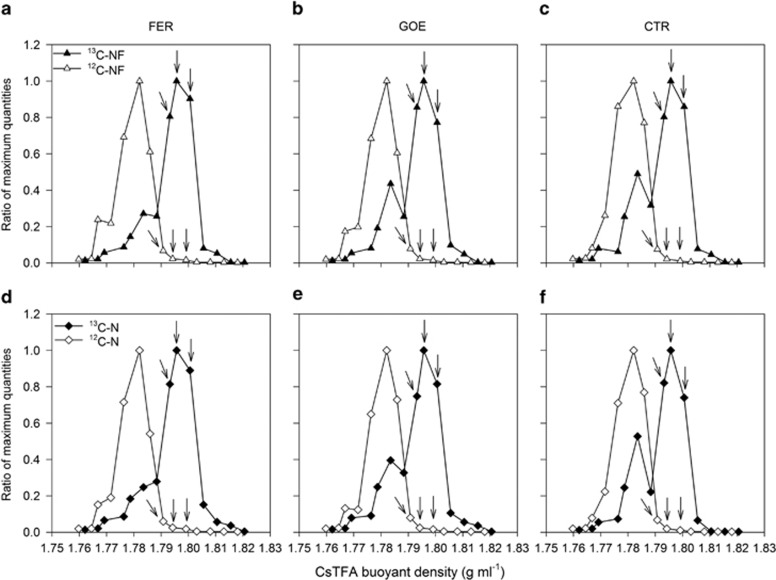
Quantitative distribution of density-resolved bacterial 16S rRNAs obtained from non-fertilized (NF; **a**–**c**) and N-fertilized (N; **d**–**f**) soil slurries treated with ferrihydrite (FER; **a** and **d**), goethite (GOE; **b** and **e**) and control (CTR; **c** and **f**) after 4-day anoxic incubation with either labeled (^13^C) or unlabeled (^12^C) acetate as the substrate. Bacterial template distribution within rRNA gradient fractions was quantified with real-time reverse transcription PCR. The normalized data are the ratio of the copy number in each gradient fraction to the maximum quantities from each treatment. The rRNA fractions subjected to pyrosequencing analysis are marked with arrows.

For both soils, the gradients in all unlabeled treatments after 4 days clearly showed peaks of bacterial and archaeal rRNA in a ‘light' RNA fraction (BD of 1.782 g ml^−1^; Figure 3 and Supplementary Figure 1). In contrast, the bulk of bacterial rRNA in all labeled treatments had apparently shifted toward ‘heavier' BDs and now banded between 1.791 and 1.806 g ml^−1^. The amounts of bacterial rRNA were considerably higher in the ‘heavy' RNA fractions from the labeled treatments than those from the corresponding unlabeled treatments. This suggests that the targeted bacterial populations were successfully labeled during microcosm incubations with ^13^C-acetate. However, an enrichment of archaeal rRNA in the ‘heavy' fractions was not detected in all labeled treatments (Supplementary Figure 1).

### Bacterial and archaeal community dynamics in density gradient fractions of rRNA

For both soils, pair-wise comparisons of bacterial rRNAs in the ‘heavy' fractions between the labeled and unlabeled treatments showed distinct community composition (Supplementary Figures 2–4), further suggesting the labeling of targeted bacterial populations during microcosm incubations. Furthermore, in all labeled treatments, the bacterial T-RFLP fingerprinting patterns in the ‘heavy' fractions in the NF soil were highly distinct from those in the N soil (Supplementary Figures 2–4a and c). Nevertheless, in both soils, a terminal restriction fragment of 159 bp in the ‘heavy' fractions significantly increased in relative abundance following the addition of Fe(III) oxyhydroxides (Supplementary Figures 2–3a and c). In the highest density fraction (BDs of 1.811 and 1.816 g ml^−1^ for NF and N soils, respectively), this terminal restriction fragment became predominant in the presence of Fe(III) oxyhydroxides in both soils, and its relative abundance in the N soil (24–46%) was much higher than that in the NF soil (11–22%).

For the archaeal T-RFLP fingerprinting patterns, a terminal restriction fragment of 187 bp was exclusively dominant throughout the density fractions in both labeled and unlabeled treatments (Supplementary Figures 5–7), and no significant difference was found in its relative abundance between the labeled and unlabeled treatments, suggesting that archaeal populations were not labeled during microcosm incubations.

### Phylogenetic identification of microorganisms assimilating ^13^C-acetate

Based on the patterns of quantitative distribution and T-RFLP fingerprints, the ‘heavy' rRNA fractions with BDs of 1.791–1.801 g ml^−1^ (fractions 5, 6 and 7) in both labeled and unlabeled treatments for both soils were selected to subject to 16S rRNA-based pyrosequencing to identify the active microbial communities represented in these rRNA fractions. The overview of pyrosequencing results of 36 rRNA fraction samples (three labeled/unlabeled treatments × three rRNA fractions per treatment × two soils) were shown in Supplementary Table 1.

**Figure 4:**
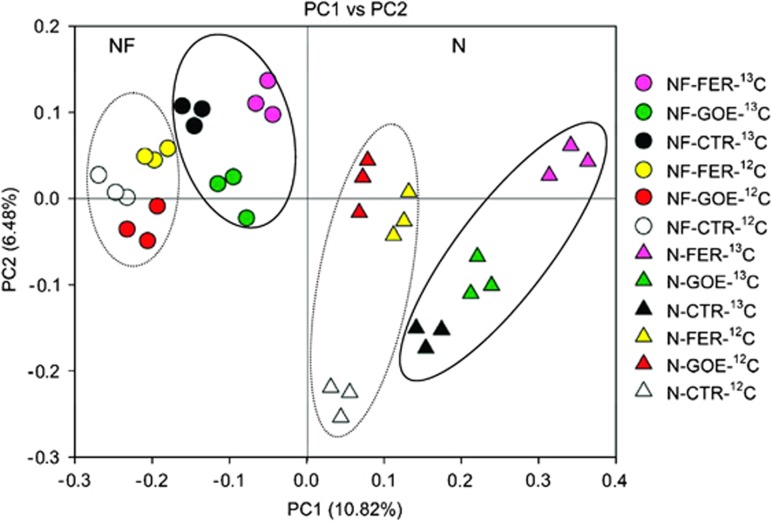
Principal coordinate analysis (PCoA) of unweighted UniFrac distances of 16S rRNA genes from 36 samples (three labeled/unlabeled treatments × three rRNA fractions per treatment × two soils). The analysis was conducted on a randomly selected subset of 3990 sequences per sample. The scatterplot is of principal coordinate 1 (PC1) vs principal coordinate 2 (PC2). The percentage of the variation in the samples described by the plotted PCs is shown on the axes. All three rRNA fractions of each treatment are represented by a single color. Ellipses in solid line denote the samples from the ^13^C-labeled treatments for NF (circles) and N (triangles) soils. Ellipses in dotted line denote the samples from the ^12^C-unlabeled treatments for NF and N soils.

We clustered these samples based on the phylogenetic lineages that they contain via the application of principal coordinate analysis to a matrix of unweighted UniFrac distances. As shown in Figure 4, three rRNA fraction samples from the same treatment grouped together. For both soils, pair-wise comparisons of samples between the labeled and unlabeled treatments showed distinct separation along principal coordinate 1 (PC1) and principal coordinate 2 (PC2), further suggesting the labeling of targeted microorganisms during anoxic incubations. In the labeled or unlabeled treatments, samples from the FER and GOE treatments were well separated from samples from the CTR treatment along PC1 and PC2. Overall, all 36 samples fell into two discrete clusters, samples from the N soil completely clustered to the right of the samples from the NF soil along PC1, even though each soil contains different incubation treatments (that is, the FER, GOE and CTR treatments).

**Figure 5:**
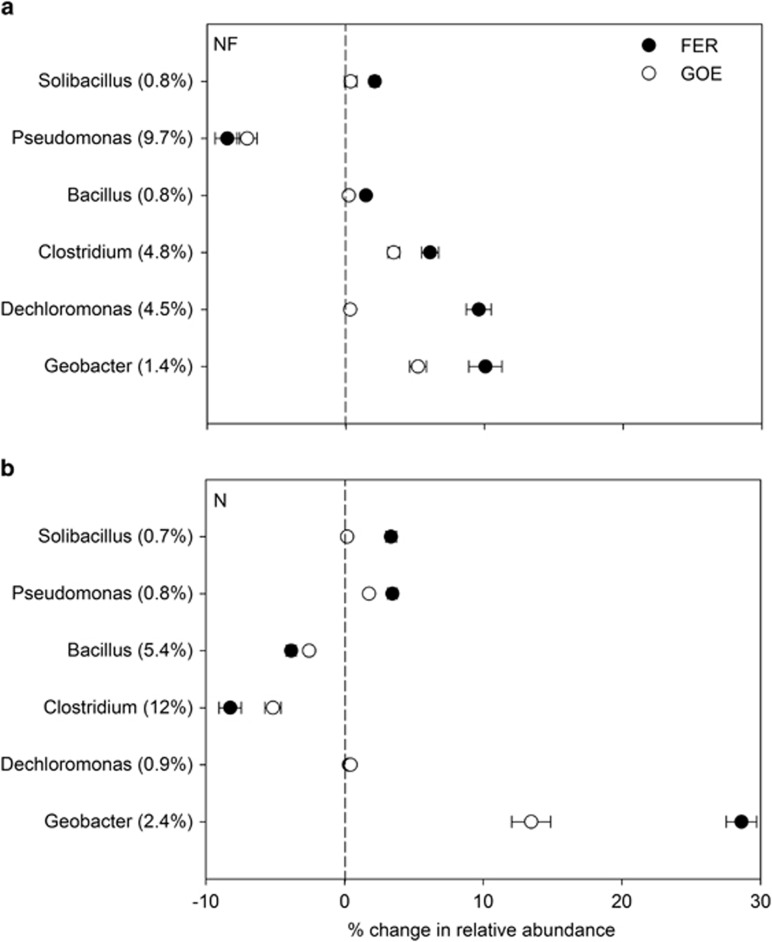
Net proportional changes in relative abundance of the potential ^13^C-acetate-assimilating bacterial populations at genus level in NF (**a**) and N (**b**) soils following the addition of ferrihydrite (FER) and goethite (GOE). The relative abundance is expressed as the average percentage of the targeted sequences to the total high-quality bacterial sequences of three rRNA fraction samples (fractions 5, 6 and 7) in each labeled treatment for each soil. *Y* axis shows the relative abundance of the targeted microorganisms in the control (CTR) treatment in each soil. The net proportional change is calculated as the difference in the relative abundance of the targeted microorganisms between the FER (or GOE) and CTR treatments in each soil. The error bars represent the standard deviations of three rRNA fraction samples.

High-quality bacterial and archaeal sequence reads were assigned to different phylogenetic taxa at genus level by ribosome database project Classifier, respectively. For the active bacterial communities, the 10 most dominant genera in each treatment were selected, and their average relative abundances were compared with those in other treatments (Supplementary Figure 8). For both soils, six genera (belonging to *Proteobacteria* and *Firmicutes*), including *Dechloromonas*, *Geobacter*, *Clostridium*, *Pseudomonas*, *Solibacillus* and *Bacillus*, had significantly (*P*<0.05) higher abundances in the labeled treatments compared with the corresponding unlabeled treatments, and thus were thought to be potential acetate-assimilating microorganisms. Among these potential ^13^C-acetate-assimilating populations, five genera including *Geobacter*, *Dechloromonas*, *Clostridium*, *Bacillus* and *Solibacillus* were stimulated by FER addition in the NF soil (Figure 5a). The relative abundances of *Geobacter* and *Solibacillus* spp. in the N soil also increased significantly (by 13- and 5.0-folds, respectively) following the addition of FER (Figure 5b), but to a greater extent compared with the NF soil (by 8.2- and 3.5-folds, respectively). Moreover, *Pseudomonas* spp. were stimulated by FER addition in the N soil. In the presence of GOE, the relative abundance of *Geobacter* spp. increased significantly (by 4.7- to 6.5-folds) in both soils, but to a lower extent compared with the FER treatment (by 8.2- to 13-folds). Furthermore, *Clostridium* spp. (by 1.7-folds) in the NF soil and *Pseudomonas* spp. (by 3.1-folds) in the N soil were enriched by GOE amendment.

For the active archaeal communities in the labeled treatments, *Methanosarcina* was the most predominant genus in both soils, and no significant difference in the relative abundance of this genus was observed between NF and N soils within each treatment (Supplementary Figure 9a). Similar trends were also detected in the unlabeled treatments for both soils (Supplementary Figure 9b).

### Phylogenetic identification of putative dissimilatory iron-reducing bacteria in field samples

**Figure 6:**
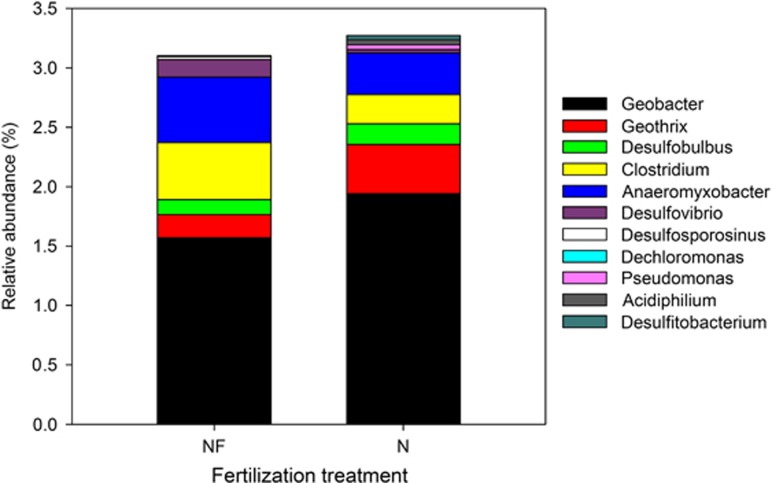
Average relative abundance of putative dissimilatory iron-reducing bacterial community structures at genus level in NF and N soils under field conditions. The abundance is expressed as the average percentage of the targeted sequences to the total high-quality bacterial sequences of samples from triplicate plots of each fertilization treatment.

Different putative dissimilatory iron-reducing bacterial community structure in original field samples detected by 16S rRNA gene based-pyrosequencing as showed that samples from the N soil were clustered together and separated by the first axis from those from the NF soil (Supplementary Figure 10). Significant (*P*<0.05) difference was observed between the NF and N soil when a non-parametric multivariate statistical test, adonis, was performed. Figure 6 summarizes the relative abundance of putative dissimilatory iron-reducing bacterial community in both soils at genus level. Six genera (belonging to *Proteobacteria*, *Acidobacteria* and *Firmicutes*), including *Geobacter*, *Geothrix*, *Desulfobulbus*, *Clostridium*, *Anaeromyxobacter* and *Desulfovibrio* were detected in both soils (Figure 6). Among these genera, the relative abundances of *Geobacter* and *Geothrix spp*. in the N soil were significantly higher than those in the NF soil, whereas *Clostridium* spp. had a significantly higher abundance in the NF compared with the N soils. In addition, *Desulfosporosinus* and *Dechloromonas* spp. were observed only in the NF soil, and *Pseudomonas*, *Desulfitobacterium* and *Acidiphilium* spp. only in the N soil.

The possible relationships between putative dissimilatory iron-reducing bacterial community structure and environmental factors were discerned with canonical correspondence analysis (Supplementary Figure 10). According to variance inflation factors with 999 Monte–Carlo permutations, three significant factors, amorphous Fe(III) oxides, total C and N, were chosen in the canonical correlation analysis biplot. The first axis, which was positively correlated with concentrations of amorphous Fe(III) oxides, total N and C, explained 33.9% of the structural variation observed, whereas the second axis explained 30.1% of the variation.

## Discussion

In this study, we investigated the shift of acetate-metabolizing iron-reducers under long-term N fertilization in a ^13^C-acetate-based SIP incubation experiment. Furthermore, the shift of putative dissimilatory iron-reducers in original field samples were also investigated by 16S rRNA gene-based pyrosequencing to examine the relevance between the results obtained from SIP incubations and those from field samples. During SIP incubations, long-term N fertilization promotes Fe(III) reduction and shifts the acetate-assimilating iron-reducing bacterial community in paddy soils. Although FER and GOE were selecting for distinct dissimilatory iron-reducing bacterial populations, the *Geobacter* spp. were identified as the most important iron-reducing bacteria and greatly stimulated by long-term N fertilization. These SIP results were supported by those obtained from the original field samples. Taken together, we demonstrate that long-term N fertilization promotes iron(III) reduction and modulates iron-reducing bacterial community in paddy soils.

### Effect of long-term N fertilization on iron reduction

During the SIP incubations, significant Fe(II) formation was found in the presence of FER and GOE, and apparently enhanced in the N-fertilized soil. This indicated that long-term N fertilization could promote this microbially mediated rather than chemical process. Furthermore, the electrons released and used for added or indigenous Fe(III) (oxyhydr)oxides reduction were calculated from the acetate consumption alone in all treatments for both soils after 4 days based on the theoretical stoichiometry, because of the absence of other volatile fatty acids (for example, propionate or butyrate) throughout the incubation (Table 3). The total amount of electrons released from the acetate was compared with that utilized to reduce amended or indigenous Fe(III) (oxyhydr)oxides. For both soils, it was estimated that exceptionally high amount of electrons flowed into Fe(III) reduction occurred in the FER treatment (74–84%) compared with the GOE (14–31%) and CTR (4.6–11%) treatments. In addition, the amount of electrons transferred to FER (234 meq e^−^) was much greater in the N soil than that in the NF soil (142 meq e^−^). These results further indicated that long-term N fertilization enhanced Fe(III) reduction in paddy soils, especially FER reduction, probably due to the shift of iron-reducing microbial community.

### Nitrogen fertilization shifts acetate-assimilating iron-reducing bacterial community

An inherent limitation associated with SIP technique is the necessity of adding a ^13^C-labeled substrate in relatively large amounts, thus greatly elevating the *in situ* availability. In our study, the ^13^C-acetate concentration added (3.3 μmol g^−1^ dry soil) was relevant to the indigenous acetate concentrations (ranging from 0.9 to 4.5 μmol g^−1^) in both soils after the pre-incubation, but about 10–50 times higher than usually observed under field conditions (Yao *et al.*, 1999; Krüger *et al.*, 2005). This may cause a discrepancy between the experimental and the actual field conditions. We minimized this discrepancy by application of highly sensitive RNA-SIP and limiting the isotope probing to a rather short time (4 days). Under these conditions, the low recovery of inorganic ^13^C in the presence of Fe(III) oxyhydroxides (Table 2) implicated that part of the ^13^C not recovered was incorporated into the rRNA. This was further validated by larger quantity of electrons transferred to Fe(III) (Table 3), and specific ^13^C assimilation into rRNA after 4-day incubation (Figure 3 and Supplementary Figures 1–7). Overall, it is apparently essential for SIP to achieve efficient labeling of rRNA, and thus the increase of acetate concentrations is a necessary compromise.

16S rRNA-based pyrosequencing results from the SIP incubations revealed that long-term N fertilization alters the structures of acetate-assimilating iron-reducing bacterial populations in the presence of Fe(III) oxyhydroxides (that is, FER and GOE) in paddy soils (Figure 5). In fact, the shifts of soil microbial communities induced by N fertilization have been found in many studies, and they might have mostly resulted from the changes in soil chemical properties (for example, pH) after long-term N input (Hallin *et al.*, 2009; Shen *et al.*, 2010; Ramirez *et al.*, 2010). In our study, one possible explanation for this interesting result could be the significant enhancement of soil labile organic C such as dissolved organic carbon under long-term N fertilization (Table 1). This might stimulate the iron-reducing bacterial community and thus promote Fe(III) reduction, given that the process of Fe(III) reduction coupled to anoxic oxidation of organic compounds has been considered as the second most important electron sink in paddy soils (Lovley and Phillips, 1986; Yao *et al.*, 1999). Another possible explanation could be the substantial increment of soil ammonium (Table 1) resulting from urea hydrolysis after long-term N fertilization. It has been suggested that in addition to organic compounds, iron-reducing microorganisms could use ammonium as an alternative electron donor for Fe(III) reduction under anoxic and reduced conditions (Chen *et al.*, 2008; Yang *et al.*, 2012).

Among the active iron-reducing bacterial populations detected, *Geobacter* spp. are of interest because of their sole respiratory (dissimilatory) metabolism type (Lovley *et al.*, 2004), and the apparent enrichment by FER and GOE addition in both soils (Figure 5), although different forms of Fe(III) oxyhydroxide were selecting for distinct populations of iron-reducing bacteria. The quantity of electron transferred to Fe(III) by dissimilatory iron-reducing microorganisms is much greater than that by fermentative iron-reducing microorganisms (Lovley, 2006). It is believed, therefore, that the dissimilatory microorganisms have a more important part in Fe(III) reduction process. Members of *Geobacter* are a well-known branch of dissimilatory iron reducers (Lovley and Phillips, 1988), they have the ability to oxidize acetate completely to carbon dioxide with Fe(III) serving as the sole electron acceptor and also have been found in many other anoxic environments (Weber *et al.*, 2006a). Intriguingly, in the presence of FER and GOE, this population had an apparently higher relative abundance in the N (16–31% of total bacterial classified sequences) than the NF soils (6.6–11%). This might explain why the amount of electron transferred to FER and GOE in the N soil was much more than that in the NF soil (Table 3).

It is also noteworthy that *Dechloromonas* spp. were detected as the most abundant iron reducers accounting for up to 14% of active bacterial communities among the FER-reducing bacteria in the NF soil (Figure 5a). Uncultured *Dechloromonas* spp. were also observed in anaerobic enrichment cultures with freshwater sediments under conditions of Fe(III) reduction and nitrate-dependent Fe(II) oxidation, but there was no direct evidence thus far about their involvement in Fe(III) reduction (Weber *et al.*, 2006b). In this study, as reducing conditions prevailed throughout the incubation, and no other electron acceptors were available, it is assumed that these novel, as-yet-uncultured, *Dechloromonas* spp. might be involved in FER reduction. However, it is not clear why the stimulation of *Dechloromonas* spp. only occurred in the NF soil treated with FER, and needs further investigations.

Our SIP results suggested the metabolic diversity of Fe(III) reduction might be far more complicated than previously appreciated. For example, *Pseudomonas*, *Clostridium* and *Bacillus* spp. have always been reported to reduce Fe(III) via fermentative metabolisms (Ottow and Glathe, 1971; Jones *et al.*, 1984; Lin *et al.*, 2007). Fe(III) reduction is only a minor pathway for electron disposal by these fermentative microorganisms, and they could not obtain any energy from this reduction process for growth (Lovley, 2006). Nevertheless, they can potentially reduce Fe(III) through respiratory metabolisms (Balashova and Zavarzin, 1980; Pollock *et al.*, 2007; Li *et al.*, 2011). Interestingly, although there was no report to date about the iron-reducing capability of the *Solibacillus* spp., they were observed in anoxic enrichment cultures containing acetate (non-fermentative substrate) with a paddy soil when FER was present as the sole electron acceptor, indicating a possible dissimilatory iron-reducing capability of this population (Yi *et al.*, 2012).

### Nitrogen fertilization shifts putative dissimilatory iron-reducing bacterial community under field conditions

Long-term N fertilization also alters putative dissimilatory iron-reducing bacterial community in the field samples (Figure 6 and Supplementary Figure 10), which supports the SIP results discussed above. The community in the N soil was positively affected by the contents of amorphous Fe(III) oxide, total C and N. The higher level of amorphous Fe(III) oxide, total C and N in the N soil compared with the NF soil might favor iron-reducing bacterial growth because of higher substrate and nutrient availability, and thus lead to a shift of putative dissimilatory iron-reducing bacterial community under field conditions.

The putative dissimilatory iron-reducing bacterial populations (belonging to *Proteobacteria*, *Acidobacteria* and *Firmicutes*) detected in the field samples were partly different from those found in the SIP incubations (belonging to *Proteobacteria* and *Firmicutes*; Figures 5 and 6). This probably resulted from the complex forms of indigenous Fe(III) (oxyhydr)oxides, relatively low availability of organic substrate such as acetate and redox fluctuations under field conditions. Among those putative iron-reducing bacterial populations detected, *Geobacter* spp. were the most abundant population in both soils, further indicating their important role in dissimilatory Fe(III) reduction in paddy soils. Moreover, the relative abundances (1.5–2.0% of the bacterial classified sequences) of this genus in both soils under field conditions were roughly equivalent to those (1.4–2.4%) detected in the CTR treatment (with ^13^C-acetate addition alone) for both soils after the SIP incubations, suggesting that although the ^13^C-acetate was added at a relatively high level during the SIP incubations as discussed above, the temporal change of SIP-labeled microbial communities could allow some extrapolation to the actual field conditions (Schwarz *et al.*, 2007). As with the SIP results, *Geobacter* spp. were also significantly stimulated by long-term N fertilization. This evidence, together with the shift of putative dissimilatory iron-reducing bacterial community under field conditions induced by long-term N fertilization as discussed above, confirms that the SIP results permit some extrapolation to the actual field conditions in our study.

## Conclusion

In summary, for the first time, our results revealed that long-term N fertilization promotes Fe(III) reduction and shifts the acetate-assimilating iron-reducing bacterial community in paddy soils using rRNA-SIP combined with pyrosequencing techniques in an incubation experiment. Although different forms of Fe(III) oxyhydroxide were selecting for distinct dissimilatory iron-reducing bacterial populations, the well-known *Geobacter* spp. were identified as active iron-reducing bacteria in the presence of FER and GOE, and greatly stimulated by long-term N fertilization. In addition to the *Geobacter* spp., some of the *Proteobacteria*- and *Firmicutes*-related bacteria not known as dissimilatory iron reducers to date may also be involved in FER or GOE reduction in different soils. These SIP results from microcosm incubations permit some extrapolation to the actual field conditions. The results of this study suggest the importance of long-term N fertilization on iron cycling in paddy soils, highlighting the complex biogeochemical interactions of element transformations than previously appreciated. The mechanism of shift of iron-reducing bacterial community induced by long-term N fertilization needs further elucidation.

## Figures and Tables

**Table 1:** Chemical properties of NF and N paddy soils

*Soil property*	*Fertilization practice*^a^	
	NF	N
pH (H_2_O)	5.3±0.02 b^b^	5.5±0.03 a
Total C mg g^−1^ soil	19±1.2 b	22±1.8 a
Total N mg g^−1^ soil	1.8±0.17 b	2.3±0.29 a
Total Fe mg g^−1^ soil	27±1.0 a	24±0.51 b
Amorphous Fe(III) oxide mg g^−1^ soil	2.3±0.13 b	3.1±0.21 a
Microbial biomass C μg g^−1^ soil	807±63 b	945±72 a
Microbial biomass N μg g^−1^ soil	63±4.3 b	86±6.7 a
Nitrate μg g^−1^ soil	2.9±0.18 a	2.8±0.16 a
Ammonium μg g^−1^ soil	24±1.7 b	52±1.5 a
Dissolved organic C μg g^−1^ soil	89±4.2 b	106±5.9 a
Dissolved organic N μg g^−1^ soil	22±1.0 b	26±1.4 a
Acetate μmol g^−1^ soil	0.28±0.01 b	0.77±0.05 a

Abbreviations: N, nitrogen fertilized; NF, non-fertilized.

a Control without fertilizers (NF); nitrogen fertilizer as urea (N).

b Mean±standard deviation (*n*=3). Values within the same row followed by the same letter do not differ at *P*<0.05.

**Table 2:** The fate of ^13^C-acetate after 4-day anoxic incubations of NF and N soil slurries in the treatments with FER, GOE and CTR

	*FER*		*GOE*		*CTR*	
	*NF*	*N*	*NF*	*N*	*NF*	*N*
^13^C from acetate added (μmol)	60	60	60	60	60	60
Gaseous ^13^CH_4_ (μmol)	9.9	7.6	13	9.5	14	11
Gaseous ^13^CO_2_ (μmol)	2.6	4.1	2.7	4.7	3.1	5.0
Dissolved ^13^CO_2_^a^ (μmol)	3.3 (7.7)^b^	8.0 (7.9)	2.8 (7.6)	7.3 (7.8)	2.6 (7.5)	5.1 (7.6)
^13^C recovery^c^ (%)	27	33	31	36	33	35

Abbreviations: CTR, control; FER, ferrihydrite; GOE, goethite; N, N-fertilized; NF, non-fertilized.

a Dissolved ^13^CO_2_ components including carbonic acid, bicarbonate and carbonate were estimated by using the gaseous ^13^CO_2_ concentration and the pH of the soil slurries after the incubation.

b The pH of soil slurries in each treatment after the incubation is presented in the parenthesis.

c ^13^C recovery is the total proportion of ^13^C from gaseous and dissolved products to the ^13^C from acetate added.

**Table 3:** The electron balance calculated after 4-day anoxic incubations of NF and N soil slurries in the treatments with FER, GOE and CTR

	*FER*		*GOE*		*CTR*	
	*NF*	*N*	*NF*	*N*	*NF*	*N*
Fe(II) formation (μmol)	142	234	23	60	6.0	15
Acetate consumption (μmol)	24	35	21	24	16	17
Electron production (meq e^−^)	192	279	164	194	130	136
Electron flowed into Fe(III) reduction (meq e^−^)	142	234	23	60	6.0	15
Percentage of electron flowed into Fe(III) reduction (%)^a^	74	84	14	31	4.6	11

Abbreviations: CTR, control; FER, ferrihydrite; GOE, goethite; N, N-fertilized; NF, non-fertilized.

a Indicates the proportion of the amount of electron flowed into amended or indigenous Fe(III) to the total amount of electron production.
