# Supplementary material for: Long-term nitrogen fertilization of paddy soil shifts iron-reducing microbial community revealed by RNA-13C-acetate probing coupled with pyrosequencing
Source: ISME J. 2014 Aug 29;9(3):721–34. doi: 10.1038/ismej.2014.159 (PMC4331580; doi:10.1038/ismej.2014.159)
Supplement: Supplementary Information [file ismej2014159x1.doc]

**Supplementary Online Material for**

Long-term nitrogen fertilization of paddy soil shifts iron-reducing microbial community revealed by RNA-^13^C-acetate probing coupled with pyrosequencing

Long-Jun Ding^1, 2^, Jian-Qiang Su^3^, Hui-Juan Xu^2, 3^, Zhong-Jun Jia^4^ and Yong-Guan Zhu^1, 3*^

^*^Correspondence should be addressed to Yong-Guan Zhu at

^1^State Key Laboratory of Urban and Regional Ecology,

Research Center for Eco-Environmental Sciences,

Chinese Academy of Sciences,

Shuangqing Road, No. 18, Haidian District, Beijing 100085, China

E-mail: [ygzhu@rcees.ac.cn](mailto:ygzhu@rcees.ac.cn)

Phone: 86-592-6190997

Fax: 86-592-6190997

This SOM file contains:

Supplementary Materials and Methods

Supplementary Table Legends

Supplementary Figure Legends

Supplementary Table S1

Supplementary References

Supplementary Figures S1 to S10

**Supplementary Materials and Methods**

**Site description and soil sampling**

The long-term fertilization experiment site (established in 1990) was located in the Taoyuan Agro-ecosystem Research Station (28°55’N, 111°27’E), central Hunan Province of China. This region has a central-north subtropical monsoon moist climate with an annual mean precipitation of 1,448 mm and annual mean temperature of 16.5°C. The paddy soil in this station was developed from quaternary red clay and classified as a waterloggogenic paddy soil. The cropping system was double-cropped rice plus green manure annually since 1990.

In our study, surface soil samples (0–20 cm depth) were collected in September (autumn) 2010 from the following two treatments: control without fertilizers (NF), and nitrogen fertilizer as urea (182 kg N ha^-1^ per year; N). Each fertilization treatment had triplicate plots which were randomly arranged in the field. All plots were sampled independently, and within each plot (33 square meter), ten random soil cores were taken, homogenized by mixing, sealed in sterile plastic bags and transported on ice to the laboratory within 12 hours.

**Soil slurry RNA extraction**

Prior to nucleic acid extraction, all solutions and glassware were rendered RNase-free by diethyl pyrocarbonate (DEPC) treatment, and only certified RNase-free plasticware was used. 500 μl of soil slurry was placed into a 2.0-ml skirted screw cap microtube containing 1.0 g of glass beads (0.17- to 0.18-mm diameter), 500 μl of hexadecyltrimethylammonium bromide (CTAB) extraction buffer, and 500 μl of phenol-chloroform-isoamyl alcohol [25:24:1 (v/v/v)] (pH 8.0). CTAB extraction buffer was prepared by adding equal volumes of 10% (w/v) CTAB in 0.7 M NaCl to 240 mM potassium phosphate buffer, pH 8.0. Samples were lysed in a FastPrep FP120 bead beating system (Bio-101, Vista, California) at 5.5 m s^-1^ for 30 s. Glass beads, soil and cell debris were pelleted by centrifugation at 16,000 × g for 5 min at 4°C, and the supernatant was transferred to a new micro-centrifuge tube. The pellet was resuspended in 500 μl of CTAB extraction buffer and 500 μl of phenol-chloroform-isoamyl alcohol [25:24:1 (v/v/v)] (pH 8.0), and the lysis procedure was repeated as described above. The supernatants of the two lysis treatments were pooled and extracted with chloroform-isoamyl alcohol [24:1 (v/v)]. The resulting aqueous phase was subsequently mixed with 2 volumes of 30% (w/v) polyethelene glycol 6000 (Fluka BioChemika)-1.6 M NaCl, incubated at room temperature for 2 h, and centrifuged for 10 min at 18,000 × g and 4°C. The total nucleic acid pellets were then washed with precooled 70% (v/v) ethanol, air dried, and resuspended in 50 μl of Tris-EDTA buffer (pH 7.4). For the removal of co-extracted DNA, the total nucleic acids were treated with RNase-free DNase (Fermentas, USA) according to the manufacture’s instructions. The DNase-treated rRNA sample was confirmed by electrophoresis on a 1.2% agarose gel.

**Statistical analysis**

SPSS (version 16.0) software was used to perform standard statistical tests, including one-way and two-way analysis of variance (ANOVA), on the soil biogeochemical and taxonomic data.

For 16S rRNA-based pyrosequencing, differences in microbial community composition were analyzed using the phylogeny-based unweighted UniFrac distance metric. This analysis determines the phylogenetic distance among microbial communities in a phylogenetic tree, and therefore provides a measure of similarity among microbial communities present in different samples (Lozupone and Knight, 2007). Average relative abundance data of predominant genus-level taxonomy in each treatment for each soil were transformed [log_2_(x+1)] and then served as input for the R PhyloTemp function (Campbell *et al*., 2010). The resulting heat map shows the relative abundance (log2 scale) of predominant genus-level assignment across the individual libraries distinctly.

For 16S rRNA gene-based pyrosequencing, a non-parametric multivariate statistical test, adonis, was conducted to examine the variation in putative dissimilatory iron-reducing bacterial community composition between NF and N soils. Canonical correspondence analysis (CCA) was performed to find out which environmental factors are important in shaping putative dissimilatory iron-reducing bacterial community. Twelve environmental factors (see Table 1) were chosen to analyze their contribution to the variation of putative dissimilatory iron-reducing bacterial community. A variance test of significance and envfit function with 999 Monte Carlo permutations were used to remove environmental factors which did not contribute significantly to the putative dissimilatory iron-reducing bacterial community variance.

**Supplementary Table Legends**

**Table S1.** Overview of pyrosequencing results using universal primer of 515F-907R targeting the 16S rRNA genes of microbial community in the selected fractionated rRNA.

**Supplementary Figure Legends**

**Figure S1.** Quantitative distribution of density-resolved archaeal 16S rRNAs obtained from non-fertilized (NF; **a–c**) and N-fertilized (N; **d–f**) soil slurries treated with ferrihydrite (FER; **a** and **d**), goethite (GOE; **b** and **e**) and control (CTR; **c** and **f**) after 4-day anoxic incubation with either labeled (^13^C) or unlabeled (^12^C) acetate as the substrate. Archaeal template distribution within rRNA gradient fractions was quantified with real-time reverse transcription-PCR. The normalized data are the ratio of the copy number in each gradient fraction to the maximum quantities from each treatment.

**Figure S2.** T-RFLP fingerprints of density-resolved bacterial 16S rRNAs obtained from non-fertilized (NF; **a** and **b**) and N-fertilized (N; **c** and **d**) soil slurries in both labeled (**a** and **c**) and unlabeled treatments (**b** and **d**) after 4-day anoxic incubation with ferrihydrite addition (FER treatment). The cesium trifluoroacetate buoyant densities (g ml^-1^) of the fractions are given in brackets. The T-RF size is shown in base pairs. The same below.

**Figure S3.** T-RFLP fingerprints of density-resolved bacterial 16S rRNAs obtained from non-fertilized (NF; **a** and **b**) and N-fertilized (N; **c** and **d**) soil slurries in both labeled (**a** and **c**) and unlabeled treatments (**b** and **d**) after 4-day anoxic incubation with goethite addition (GOE treatment).

**Figure S4.** T-RFLP fingerprints of density-resolved bacterial 16S rRNAs obtained from non-fertilized (NF; **a** and **b**) and N-fertilized (N; **c** and **d**) soil slurries in both labeled (**a** and **c**) and unlabeled treatments (**b** and **d**) after 4-day anoxic incubation without any iron(III) oxyhydroxide addition (CTR treatment).

**Figure S5.** T-RFLP fingerprints of density-resolved archaeal 16S rRNAs obtained from non-fertilized (NF; **a** and **b**) and N-fertilized (N; **c** and **d**) soil slurries in both labeled (**a** and **c**) and unlabeled treatments (**b** and **d**) after 4-day anoxic incubation with ferrihydrite addition (FER treatment).

**Figure S6.** T-RFLP fingerprints of density-resolved archaeal 16S rRNAs obtained from non-fertilized (NF; **a** and **b**) and N-fertilized (N; **c** and **d**) soil slurries in both labeled (**a** and **c**) and unlabeled treatments (**b** and **d**) after 4-day anoxic incubation with goethite addition (GOE treatment).

**Figure S7.** T-RFLP fingerprints of density-resolved archaeal 16S rRNAs obtained from non-fertilized (NF; **a** and **b**) and N-fertilized (N; **c** and **d**) soil slurries in both labeled (**a** and **c**) and unlabeled treatments (**b** and **d**) after 4-day anoxic incubation without any iron(III) oxyhydroxide addition (CTR treatment).

**Figure S8.** Heat map of the 10 most abundant genera in each treatment for each soil. The color intensity (log2 scale) in each cell indicates the relative abundance of a genus in a treatment with legend presented at the top of the figure. The relative abundance is expressed as the average percentage of the targeted sequences to the total high-quality bacterial sequences of three rRNA fraction samples (fractions 5, 6 and 7) in each treatment for each soil. The specific genera in red denote significantly (*P* < 0.05) higher percentages in the labeled treatments compared to the corresponding unlabeled treatments.

**Figure S9.** Average relative abundance of the archaeal community structures at genus level in the labeled (**a**) and unlabeled (**b**) treatments for NF and N soils in treatments with ferrihydrite (FER), goethite (GOE) and control (CTR). The relative abundance is expressed as the average percentage of the targeted sequences to the total high-quality archaeal sequences of three heavy rRNA fraction samples (fractions 5, 6 and 7) in each treatment for each soil. Other refers to the taxa with a maximum abundance of < 1% in any sample.

**Figure S10.** Canonical correspondence analysis (CCA) compares the putative dissimilatory iron-reducing bacterial community structure and environmental factors (arrows), including amorphous Fe(III) oxides (AmoFe), total C (TC) and N (TN). Environmental factors were selected based on significance calculated from individual CCA results and variance inflation factors (VIFs) calculated during CCA. The percentage of variation explained by each axis is shown.

Table S1. Overview of pyrosequencing results using universal primer of 515F-907R targeting the 16S rRNA genes of microbial community in the selected fractionated rRNA.

| rRNA gradient fraction^1^ | High-quality read number | | | | | | | | | | | | |
| --- | --- | --- | --- | --- | --- | --- | --- | --- | --- | --- | --- | --- | --- |
|  | ^13^C-labeled treatment | | | | | |  | ^12^C-unlabeled treatment | | | | | |
|  | FER | | GOE | | CTR | |  | FER | | GOE | | CTR | |
|  | NF | N | NF | N | NF | N |  | NF | N | NF | N | NF | N |
| Fraction-7 | 4450 | 5800 | 4365 | 6576 | 4440 | 7581 |  | 5784 | 5636 | 6667 | 6210 | 6345 | 8314 |
| Fraction-6 | 4554 | 5581 | 3990 | 6414 | 6350 | 7311 |  | 5340 | 5256 | 5142 | 7470 | 6484 | 7811 |
| Fraction-5 | 5598 | 5506 | 5770 | 5943 | 5238 | 6612 |  | 4808 | 6996 | 5987 | 7745 | 5210 | 8226 |
| Average | 4867 | 5629 | 4708 | 6311 | 5343 | 7168 |  | 5311 | 5963 | 5932 | 7142 | 6013 | 8117 |
| Subtotal | 14602 | 16887 | 14125 | 18933 | 16028 | 21504 |  | 15932 | 17888 | 17796 | 21425 | 18039 | 24351 |
| Total | 217510 | | | | | | | | | | | | |

^1^ Indicates that rRNA gradient fractions with different buoyant densities selected for pyrosequencing, and the smaller the number, the heavier the fractionated rRNA.

**Supplementary References**

Campbell BJ, Polson SW, Hanson TE, Mack MC, Schuur EAG. (2010). The effect of nutrient deposition on bacterial communities in Arctic tundra soil. *Environ Microbiol* **12**: 1842–1854.

Lozupone CA, Knight R. (2007). Global patterns in bacterial diversity. *Proc Natl Acad Sci USA* **104**: 11436–11440.


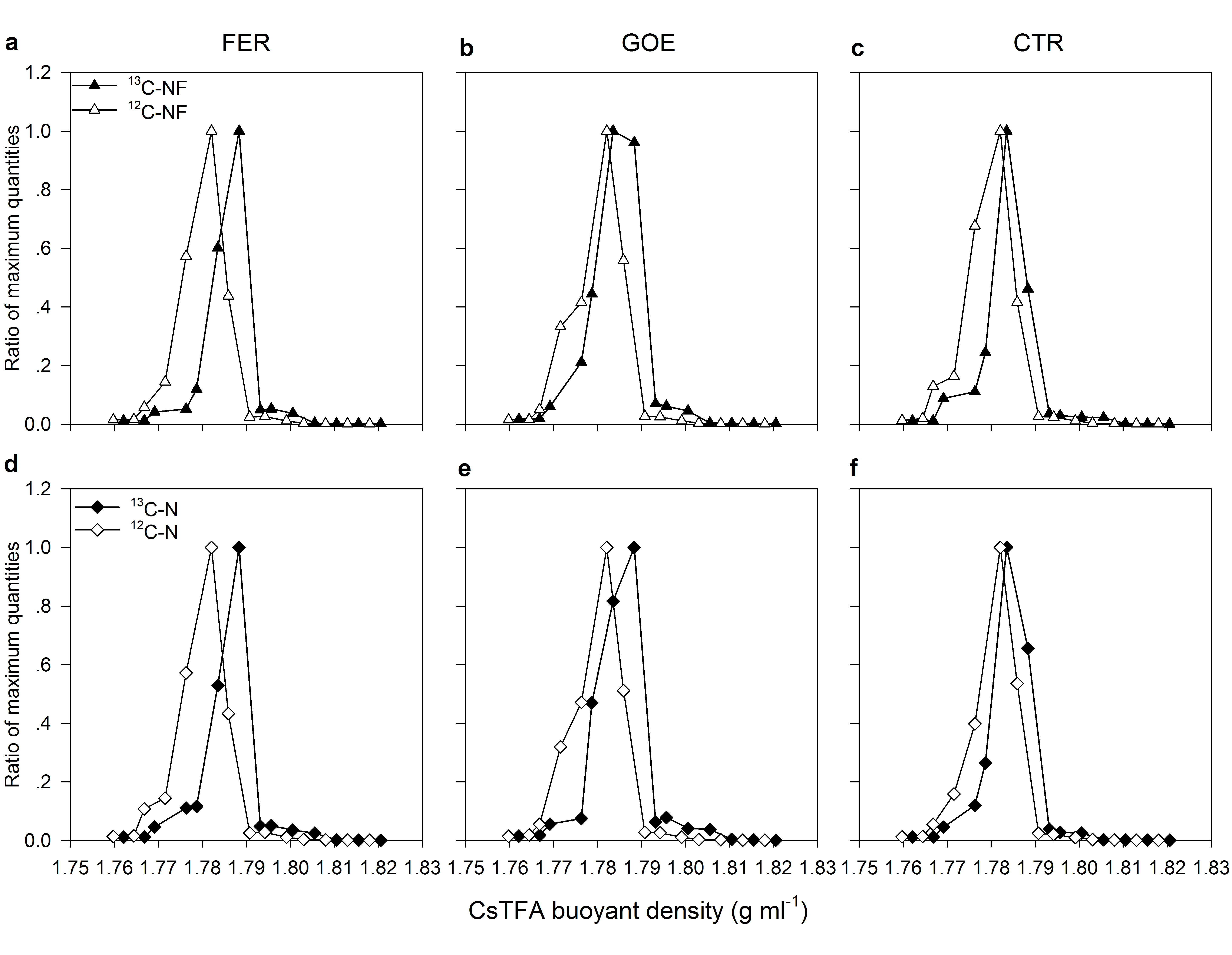


**Figure S1.** Quantitative distribution of density-resolved archaeal 16S rRNAs obtained from non-fertilized (NF; **a–c**) and N-fertilized (N; **d–f**) soil slurries treated with ferrihydrite (FER; **a** and **d**), goethite (GOE; **b** and **e**) and control (CTR; **c** and **f**) after 4-day anoxic incubation with either labeled (^13^C) or unlabeled (^12^C) acetate as the substrate. Archaeal template distribution within rRNA gradient fractions was quantified with real-time reverse transcription-PCR. The normalized data are the ratio of the copy number in each gradient fraction to the maximum quantities from each treatment.


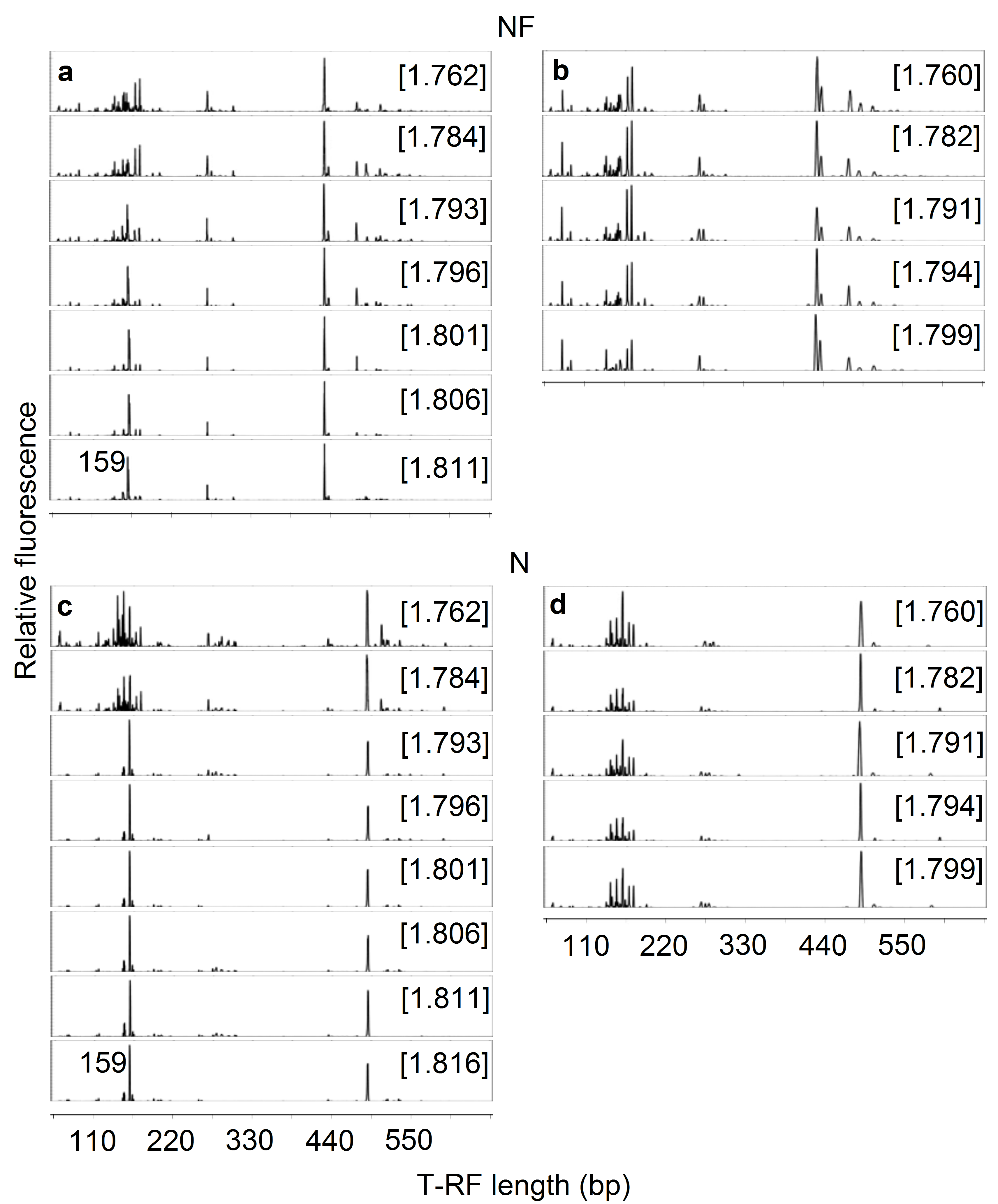


**Figure S2.** T-RFLP fingerprints of density-resolved bacterial 16S rRNAs obtained from non-fertilized (NF; **a** and **b**) and N-fertilized (N; **c** and **d**) soil slurries in both labeled (**a** and **c**) and unlabeled treatments (**b** and **d**) after 4-day anoxic incubation with ferrihydrite addition (FER treatment). The cesium trifluoroacetate buoyant densities (g ml^-1^) of the fractions are given in brackets. The T-RF size is shown in base pairs. The same below.


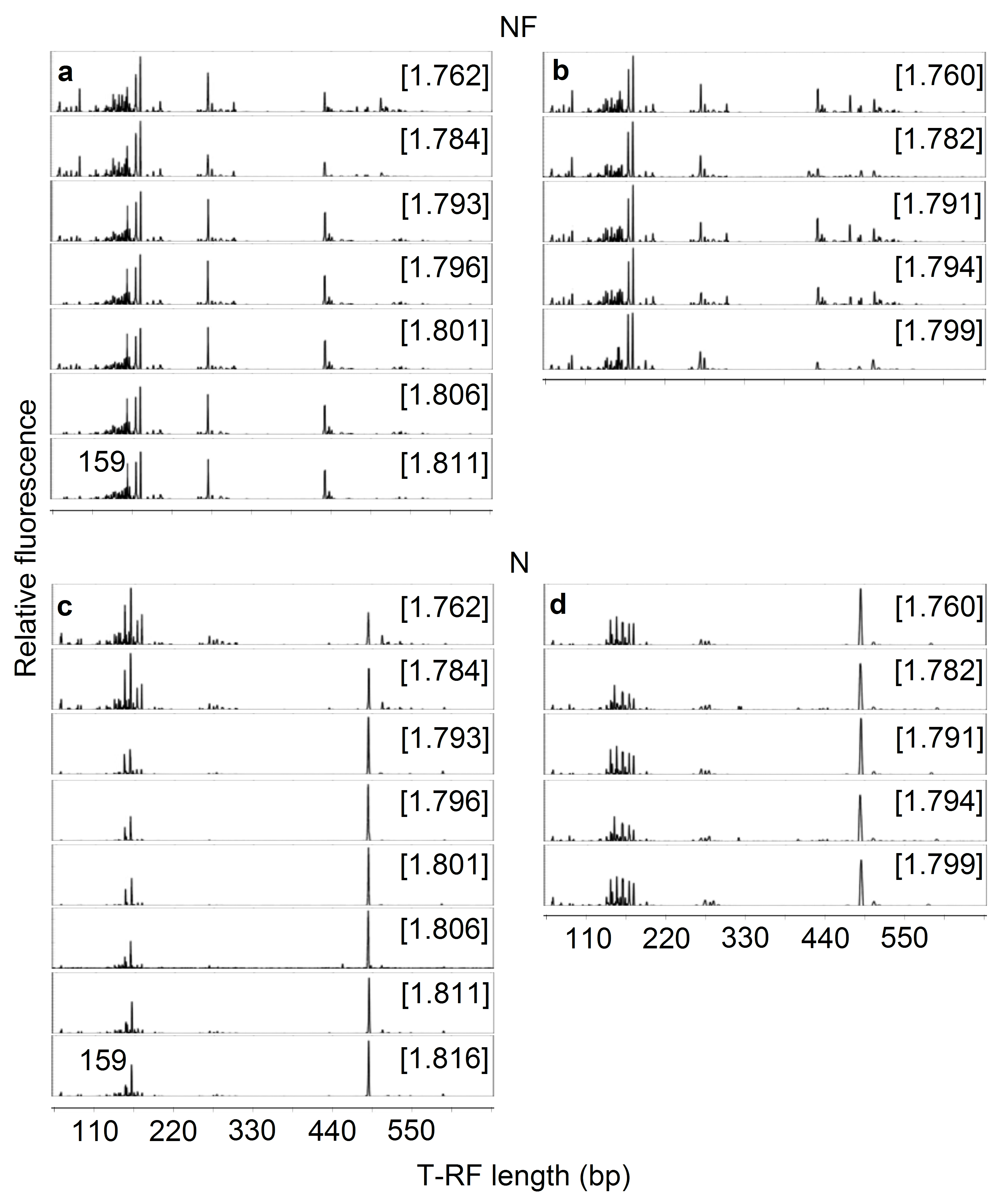


**Figure S3.** T-RFLP fingerprints of density-resolved bacterial 16S rRNAs obtained from non-fertilized (NF; **a** and **b**) and N-fertilized (N; **c** and **d**) soil slurries in both labeled (**a** and **c**) and unlabeled treatments (**b** and **d**) after 4-day anoxic incubation with goethite addition (GOE treatment).

**
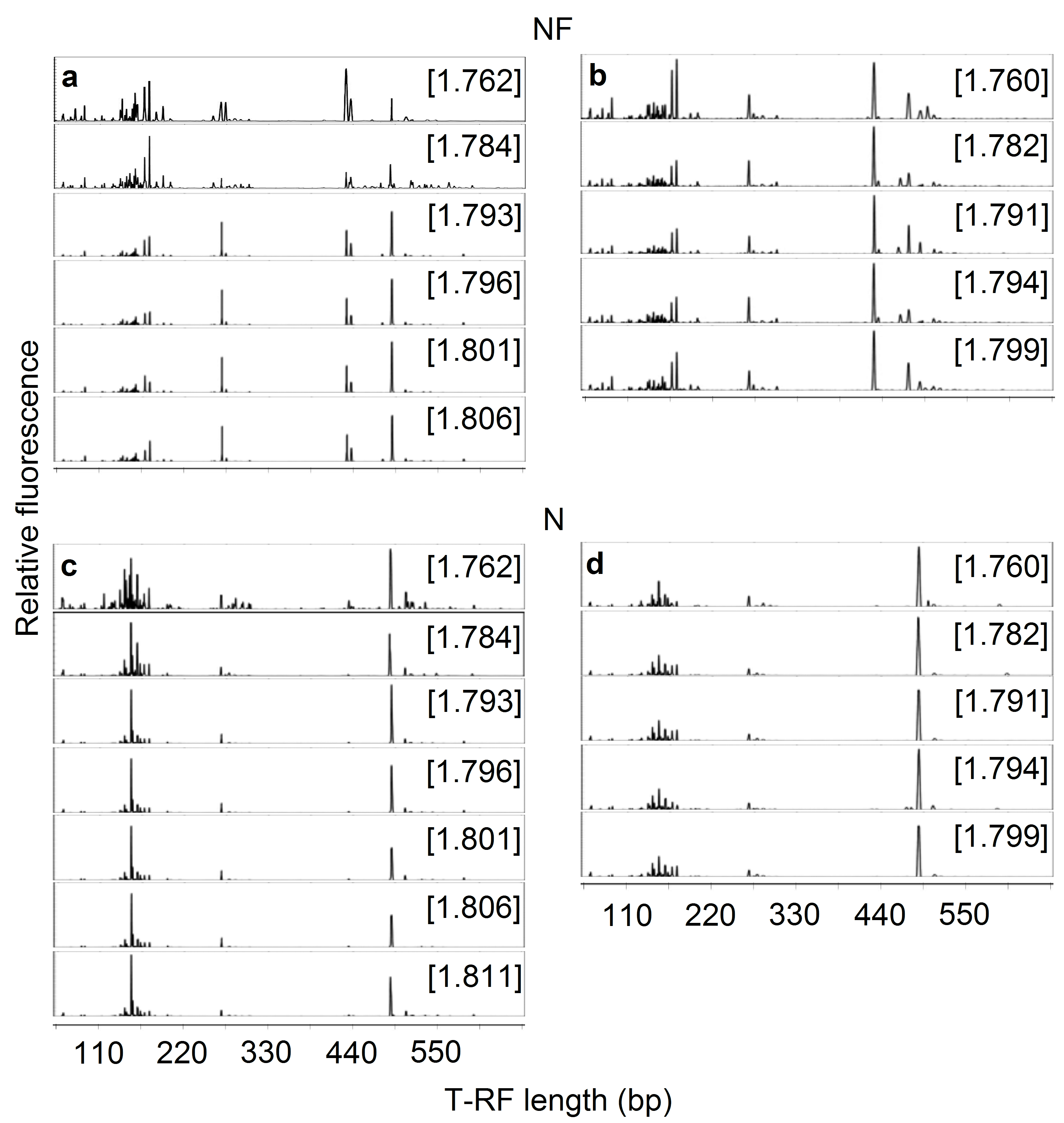
**

**Figure S4.** T-RFLP fingerprints of density-resolved bacterial 16S rRNAs obtained from non-fertilized (NF; **a** and **b**) and N-fertilized (N; **c** and **d**) soil slurries in both labeled (**a** and **c**) and unlabeled treatments (**b** and **d**) after 4-day anoxic incubation without any iron(III) oxyhydroxide addition (CTR treatment).


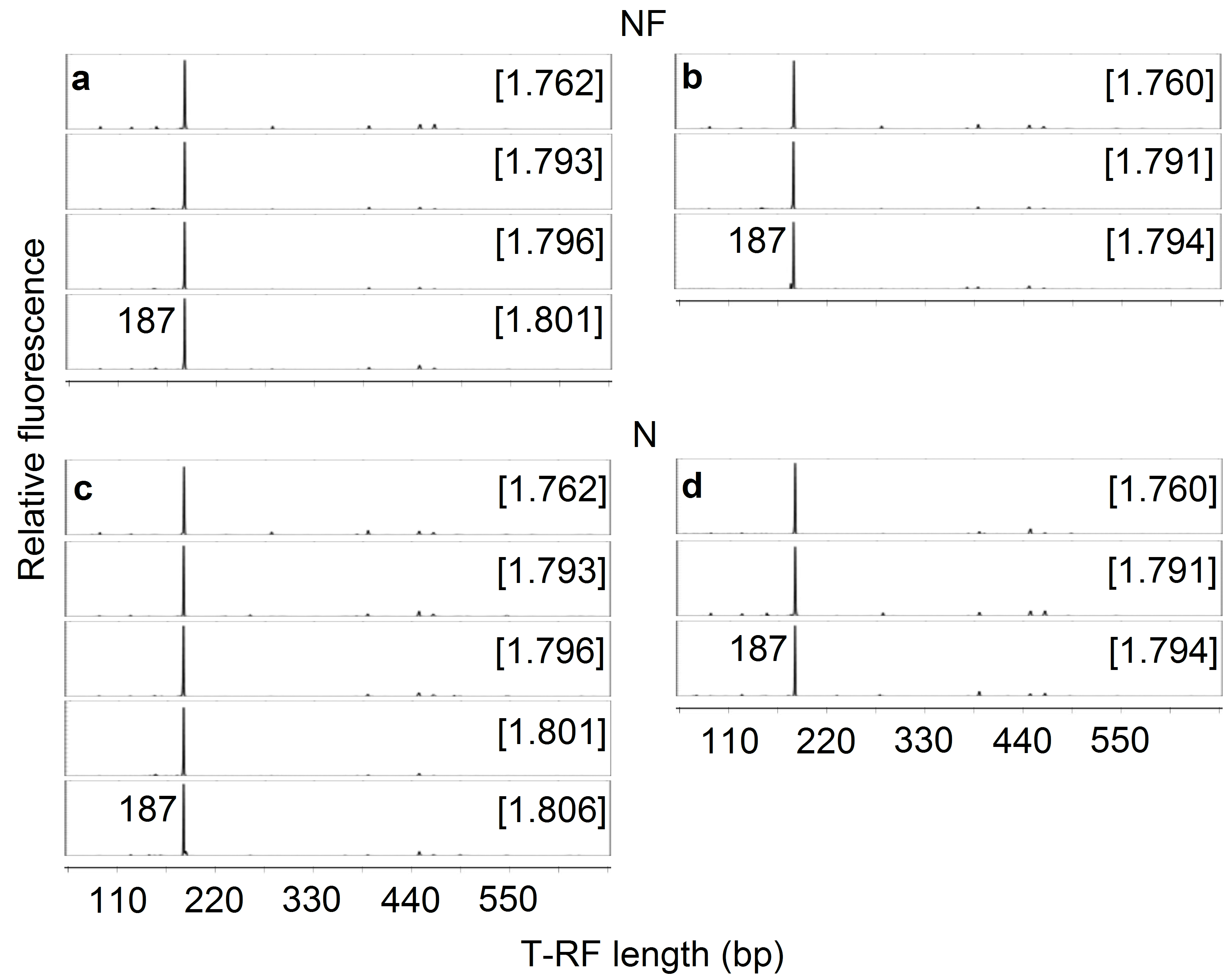


**Figure S5.** T-RFLP fingerprints of density-resolved archaeal 16S rRNAs obtained from non-fertilized (NF; **a** and **b**) and N-fertilized (N; **c** and **d**) soil slurries in both labeled (**a** and **c**) and unlabeled treatments (**b** and **d**) after 4-day anoxic incubation with ferrihydrite addition (FER treatment).


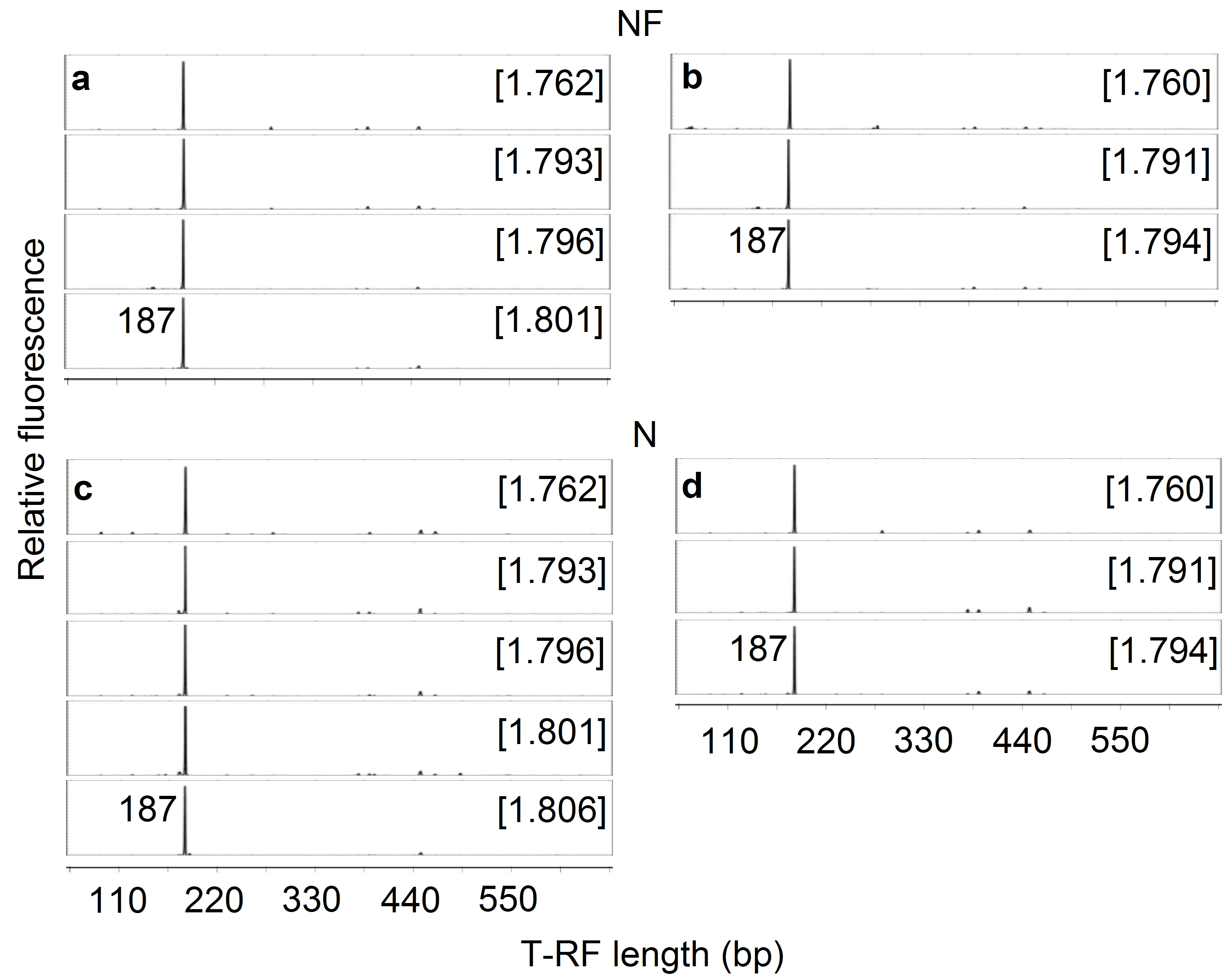


**Figure S6.** T-RFLP fingerprints of density-resolved archaeal 16S rRNAs obtained from non-fertilized (NF; **a** and **b**) and N-fertilized (N; **c** and **d**) soil slurries in both labeled (**a** and **c**) and unlabeled treatments (**b** and **d**) after 4-day anoxic incubation with goethite addition (GOE treatment).


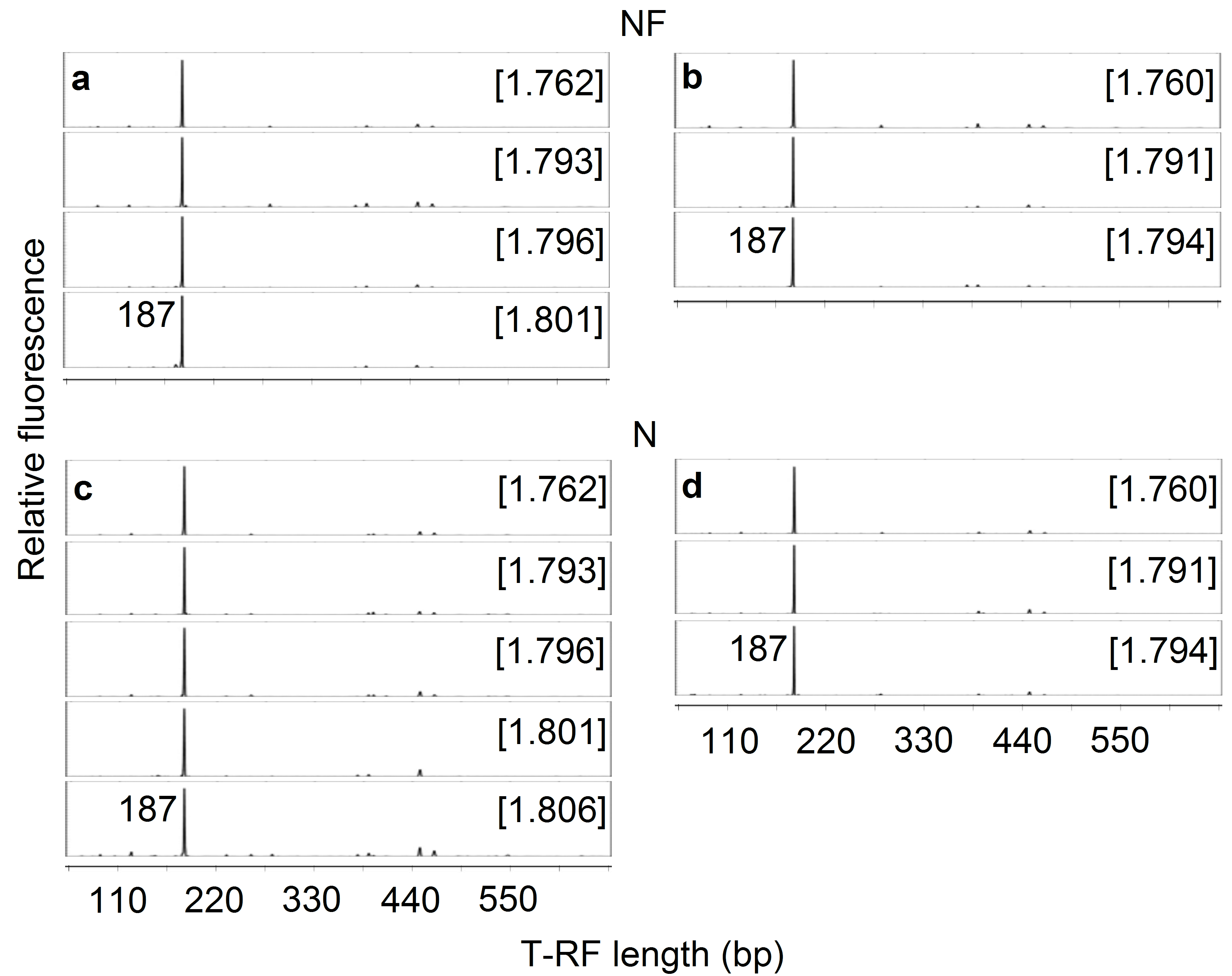


**Figure S7.** T-RFLP fingerprints of density-resolved archaeal 16S rRNAs obtained from non-fertilized (NF; **a** and **b**) and N-fertilized (N; **c** and **d**) soil slurries in both labeled (**a** and **c**) and unlabeled treatments (**b** and **d**) after 4-day anoxic incubation without any iron(III) oxyhydroxide addition (CTR treatment).


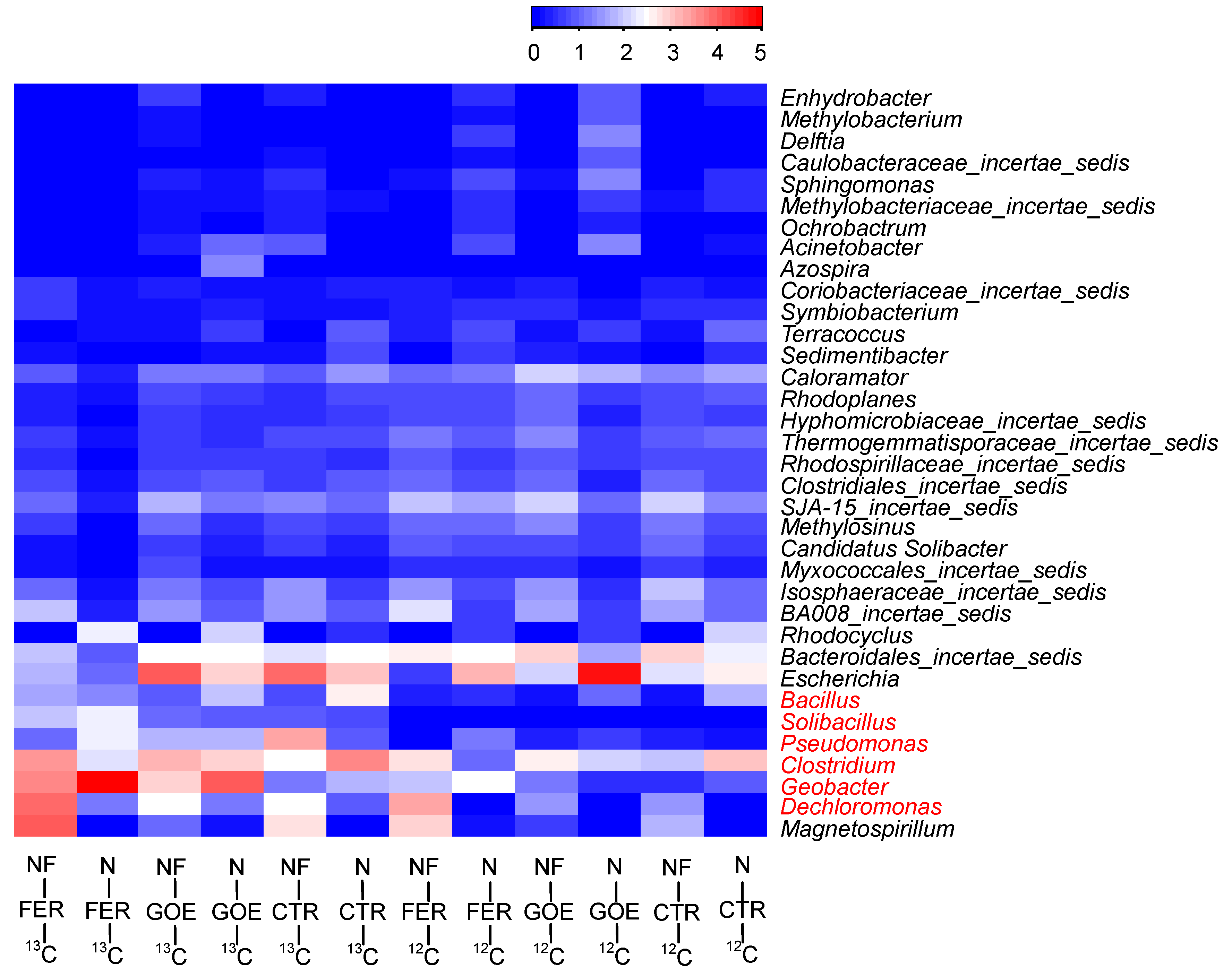


**Figure S8.** Heat map of the 10 most abundant genera in each treatment for each soil. The color intensity (log2 scale) in each cell indicates the relative abundance of a genus in a treatment with legend presented at the top of the figure. The relative abundance is expressed as the average percentage of the targeted sequences to the total high-quality bacterial sequences of three rRNA fraction samples (fractions 5, 6 and 7) in each treatment for each soil. The specific genera in red denote significantly (*P* < 0.05) higher percentages in the labeled treatments compared to the corresponding unlabeled treatments.


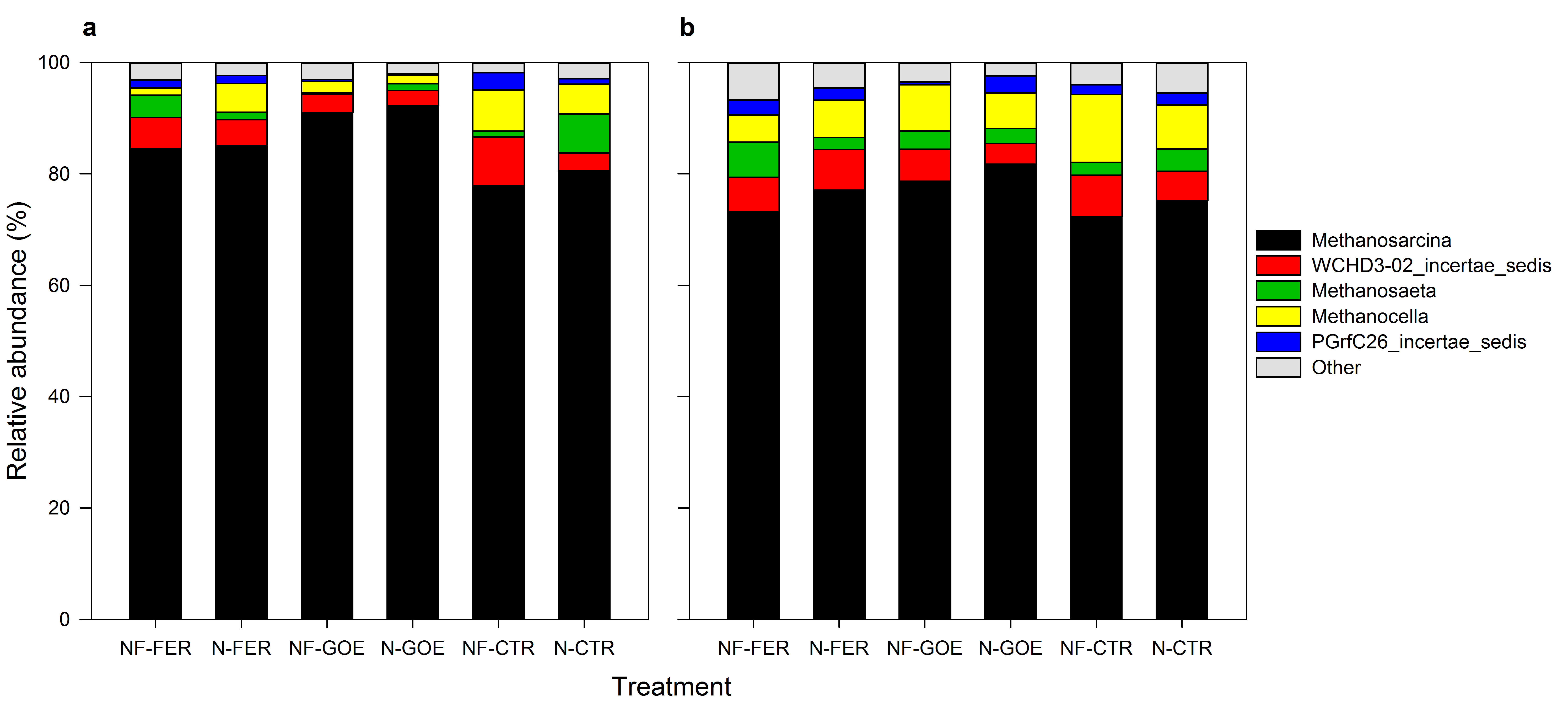


**Figure S9.** Average relative abundance of the archaeal community structures at genus level in the labeled (**a**) and unlabeled (**b**) treatments for NF and N soils in treatments with ferrihydrite (FER), goethite (GOE) and control (CTR). The relative abundance is expressed as the average percentage of the targeted sequences to the total high-quality archaeal sequences of three heavy rRNA fraction samples (fractions 5, 6 and 7) in each treatment for each soil. Other refers to the taxa with a maximum abundance of < 1% in any sample.


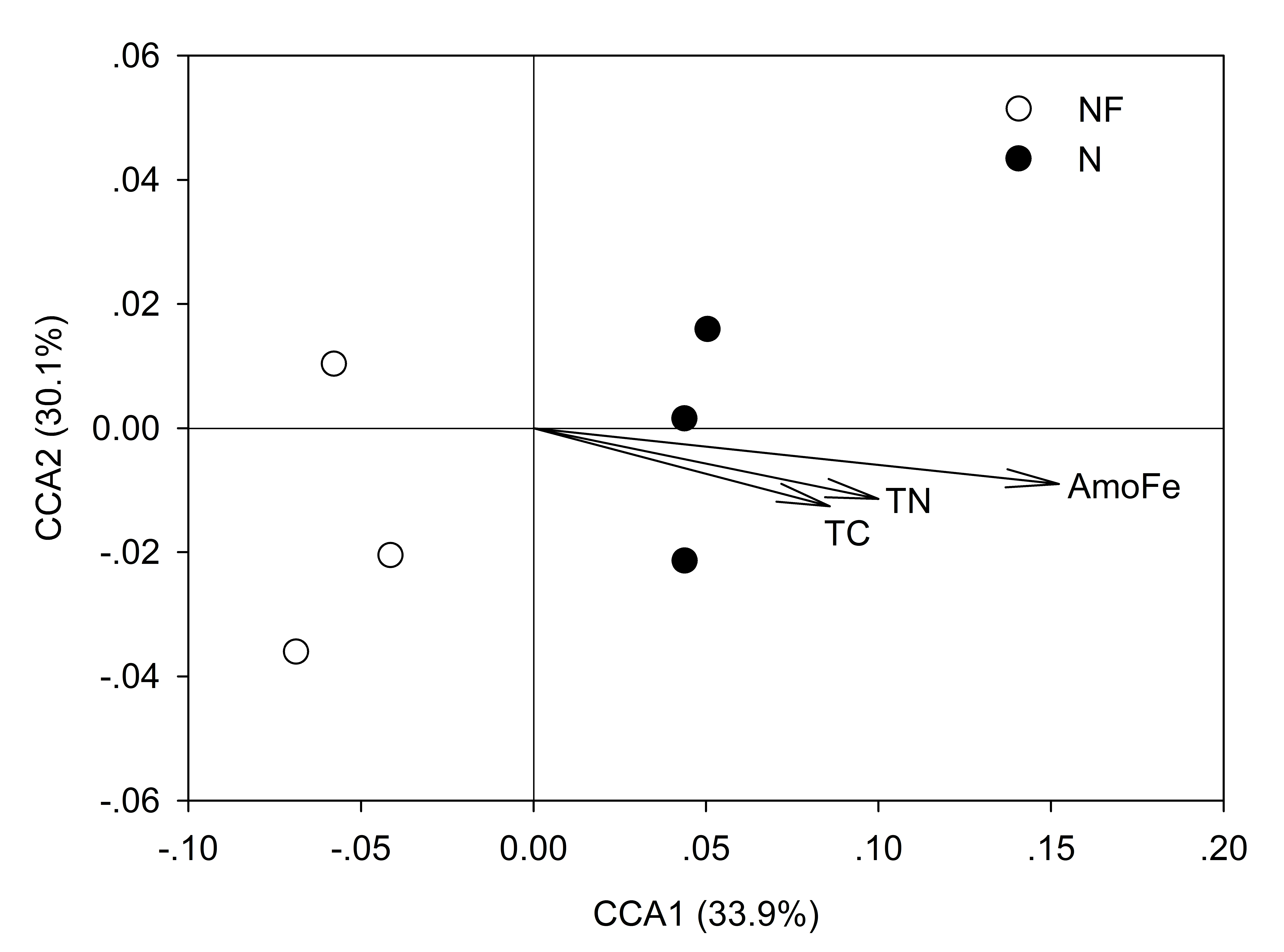


**Figure S10.** Canonical correspondence analysis (CCA) compares the putative dissimilatory iron-reducing bacterial community structure and environmental factors (arrows), including amorphous Fe(III) oxides (AmoFe), total C (TC) and N (TN). Environmental factors were selected based on significance calculated from individual CCA results and variance inflation factors (VIFs) calculated during CCA. The percentage of variation explained by each axis is shown.
